# The Influence of Health Systems on Hypertension Awareness, Treatment, and Control: A Systematic Literature Review

**DOI:** 10.1371/journal.pmed.1001490

**Published:** 2013-07-30

**Authors:** Will Maimaris, Jared Paty, Pablo Perel, Helena Legido-Quigley, Dina Balabanova, Robby Nieuwlaat, Martin Mckee

**Affiliations:** 1 London School of Hygiene and Tropical Medicine, London, United Kingdom; 2 Population Health Research Institute, Hamilton, Ontario, Canada; 3 Clinical Epidemiology & Biostatistics, McMaster University, Hamilton, Ontario, Canada; Barts and The London School of Medicine and Dentistry, United Kingdom

## Abstract

**Background:**

Hypertension (HT) affects an estimated one billion people worldwide, nearly three-quarters of whom live in low- or middle-income countries (LMICs). In both developed and developing countries, only a minority of individuals with HT are adequately treated. The reasons are many but, as with other chronic diseases, they include weaknesses in health systems. We conducted a systematic review of the influence of national or regional health systems on HT awareness, treatment, and control.

**Methods and Findings:**

Eligible studies were those that analyzed the impact of health systems arrangements at the regional or national level on HT awareness, treatment, control, or antihypertensive medication adherence. The following databases were searched on 13th May 2013: Medline, Embase, Global Health, LILACS, Africa-Wide Information, IMSEAR, IMEMR, and WPRIM. There were no date or language restrictions. Two authors independently assessed papers for inclusion, extracted data, and assessed risk of bias. A narrative synthesis of the findings was conducted. Meta-analysis was not conducted due to substantial methodological heterogeneity in included studies. 53 studies were included, 11 of which were carried out in LMICs. Most studies evaluated health system financing and only four evaluated the effect of either human, physical, social, or intellectual resources on HT outcomes. Reduced medication co-payments were associated with improved HT control and treatment adherence, mainly evaluated in US settings. On balance, health insurance coverage was associated with improved outcomes of HT care in US settings. Having a routine place of care or physician was associated with improved HT care.

**Conclusions:**

This review supports the minimization of medication co-payments in health insurance plans, and although studies were largely conducted in the US, the principle is likely to apply more generally. Studies that identify and analyze complexities and links between health systems arrangements and their effects on HT management are required, particularly in LMICs.

*Please see later in the article for the Editors' Summary*

## Abstract 2

Will Maimaris and colleagues systematically review the evidence that national or regional health systems, including place of care and medication co-pays, influence hypertension awareness, treatment, and control.

*Please see later in the article for the Editors' Summary*

## Editors' Summary

**Background:**

In 2008, one billion people, three-quarters of whom were living in low- and middle-income countries, had high blood pressure (hypertension). Worldwide, hypertension, which rarely has any symptoms, leads to about 7.5 million deaths annually from heart attacks, stroke, other cardiovascular diseases, and kidney disease. Hypertension, selected by the World Health Organization as the theme for World Health Day 2013, is diagnosed by measuring blood pressure, the force that blood circulating in the body exerts on the inside of large blood vessels. Blood pressure is highest when the heart is contracts to pump blood out (systolic blood pressure) and lowest when the heart relaxes and refills (diastolic blood pressure). Normal adult blood pressure is defined as a systolic blood pressure of less than 120 millimeters of mercury (mmHg) and a diastolic blood pressure of less than 80 mmHg (a blood pressure of less than 120/80 mmHg). A blood pressure reading of more than 140/90 mmHg indicates hypertension. Many factors affect blood pressure, but overweight people and individuals who eat fatty or salty foods are at high risk of developing hypertension.

**Why Was This Study Done?:**

Most individuals can achieve good hypertension control, which reduces death and disability from cardiovascular and kidney disease, by making lifestyle changes (mild hypertension) and/or by taking antihypertensive drugs. Yet, in both developed and developing countries, many people with hypertension are not aware of their condition and are not adequately treated. As with other chronic diseases, weaknesses in health care systems probably contribute to the inadequate treatment of hypertension. A health care system comprises all the organizations, institutions, and resources whose primary purpose is to improve health. Weaknesses in health care systems can exist at the national, regional, district, community, and household level. In this systematic review (a study that uses predefined criteria to identify all the research on a given topic), the researchers investigate how national and regional health care system arrangements influence hypertension awareness, treatment, and control. Actions that might influence hypertension care at this level of health care systems include providing treatment for hypertension at no or reduced cost, the introduction of financial incentives to healthcare practitioners for the diagnosis and treatment of hypertension, and enhanced insurance coverage in countries such as the US where people pay for health care through insurance policies.

**What Did the Researchers Do and Find?:**

The researchers identified 53 studies that analyzed whether regional or national health care systems arrangements were associated with patient awareness of hypertension, treatment of hypertension, adherence to antihypertensive medication treatment, and control of hypertension. The researchers used an established conceptual framework for health care systems and an approach called narrative synthesis to analyze the results of these studies, most of which were conducted in the US (36 studies) and other high-income countries (eight studies). Nearly all the studies evaluated the effects of health system financing on hypertension outcomes, although several looked at the effects of delivery and governance of health systems on these outcomes. The researchers' analysis revealed an association between reduced medication co-payments (drug costs that are not covered by health insurance and that are paid by patients in countries without universal free healthcare) and improved hypertension control and treatment adherence, mainly in US settings. In addition, in US settings, health insurance coverage was associated with improved hypertension outcomes, as was having a routine physician or place of care.

**What Do These Findings Mean?:**

These findings suggest that minimizing co-payments for health care and expansion of health insurance coverage in countries without universal free health care may improve the awareness, treatment, and control of hypertension. Although these findings are based mainly on US studies, they are likely to apply more generally but, importantly, these findings indicate that additional, high-quality studies are needed to unravel the impact of health systems arrangements on the management of hypertension. In particular, they reveal few studies in low- and middle-income countries where most of the global burden of hypertension lies and where weaknesses in health systems often result in deficiencies in the care of chronic diseases. Moreover, they highlight a need for studies that evaluate how aspects of health care systems other than financing (for example, delivery and governance mechanisms) and interactions between health care system arrangements affect hypertension outcomes. Without the results of such studies, governments and national and international organizations will not know the best ways to deal effectively with the global public-health crisis posed by hypertension.

**Additional Information:**

Please access these Web sites via the online version of this summary at http://dx.doi.org/10.1371/journal.pmed.1001490.

- The US National Heart Lung and Blood Institute has patient information about high blood pressure (in English and Spanish)
- The American Heart Association provides information on high blood pressure (in several languages) and personal stories about dealing with high blood pressure
- The UK National Health Service (NHS) Choices website provides detailed information for patients about hypertension and a personal story about hypertension
- The World Health Organization provides information on controlling blood pressure and on health systems (in several languages); its "A Global Brief on Hypertension" was published on World Health Day 2013
- MedlinePlus provides links to further information about high blood pressure (in English and Spanish)

## Introduction

Hypertension (HT) is common worldwide, affecting an estimated billion people, nearly three-quarters of whom live in low or middle income countries (LMICs) [1]. HT is second, after smoking, as a contributor to the Global Burden of Disease in the latest (2010) analysis [2]. In most individuals it is easily treated and controlled, with effective control reducing deaths and disability from a number of conditions, including cerebrovascular, cardiovascular, and renal disease [3]. Yet in both developed and developing countries, a significant proportion of people with HT remain unaware of their diagnosis, and of those who are aware, only a minority are treated and have their HT successfully controlled [4]. The reasons are many but, as with other chronic diseases, they include weaknesses in health systems, related to both structures and ways in which systems function [5],[6]. Health systems have been defined by the World Health Organization as “all the organizations, institutions and resources that are devoted to producing health actions” [7] and weaknesses may exist at the national, regional, district, community, and household level.

Previous systematic reviews have examined the effects of health systems interventions delivered at the community or health facility level on HT care, such as educational interventions that target providers, organisational interventions strengthening collaboration between physicians and pharmacists, and using electronic records to improve management [8]–[10]. However, we are unaware of any previous systematic review exploring the effect of actions originating at national or regional health systems level, including health policies, programs, and interventions, on HT outcomes. Actions that have been hypothesized to influence HT care include strategies for procurement of essential medications, the existence of simple national guidelines for HT management, introduction of financial incentives for health care practitioners to diagnose or treat HT, and enhanced health insurance coverage [1]. To address this gap, we systematically reviewed the literature examining the effect of national or regional health system arrangements on HT care and control, and make recommendations for future research and policy.

## Methods

**Figure 1:**
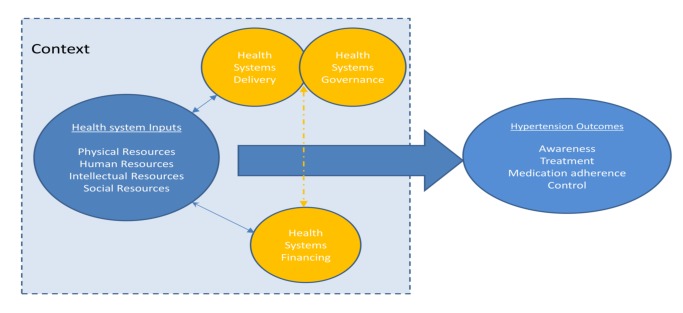
Schematic diagram of health systems conceptual framework.

A protocol for this study has been published on the PROSPERO international prospective register of systematic reviews, with the record number PROSPERO 2012:CRD42012002864 [11]. We used an established framework to illustrate the health system and its elements and guide our systematic review (Figure 1). This conceptual framework, which has been found useful in understanding the systems failings that impede effective management of non-communicable diseases [12],[13], consists of four domains relating to key system level inputs that are required for effective chronic disease care: namely, physical resources (e.g., health facilities and diagnostic equipment), human resources (e.g., trained health care workers and managers), intellectual resources (e.g., treatment guidelines), and social resources (which draws on the concept of social capital and includes organizational measures to enhance collaboration). The existence of inputs is insufficient in itself, without effective systems to finance, deliver, and govern care; and these are also reflected in the framework. All of these domains influence the impact of the health system inputs on the health care outcomes of interest, which are HT awareness, treatment, control, and antihypertensive medication adherence in this case. The framework aims to capture the complex interactions and inter-relationships that exist between the elements within a health system, acknowledging that success of health systems does not simply require a “laundry list” of building blocks (as the WHO's 2007 framework is often perceived), but requires effective integration and alignment of these inputs [14],[15]. The framework also highlights the important role that context plays in shaping the relationship between health systems inputs and outcomes, recognizing the complex adaptive nature of health systems so that changes may yield different results in different settings [16].

### Inclusion Criteria

We included studies that reported on the effects of national or regional health system level arrangements (factors, interventions, policies, or programs) on HT control and key upstream determinants of control: HT awareness, treatment, and medication adherence. Definitions of these outcomes are given in Box 1.

We included studies looking at any adult population, including general populations, populations on treatment, and studies of people with specific co-morbidities, such as diabetes.

The following types of studies were included: (1) Studies, such as controlled trials, cohort studies, and cross-sectional studies, which quantify the effects on HT outcomes of interventions, policies, or programmes, which are enacted at national or regional health system level, acting on one or more domains of the health-system. (2) Studies, such as qualitative studies, which report on the views and experiences of actors (e.g., patients, physicians, or policy makers) on national or regional health-system level barriers to HT awareness, treatment, control, or antihypertensive medication adherence. (3) Studies reporting on the impact of national or regional HT care policies or interventions that have relevance for other disease programs or for the design of the health system more broadly, such as those that require or lead to changes in primary care provision or other general aspects of the health system.

Quantitative studies were included only if they reported a measure of association between the health system arrangement under investigation and at least one of the HT outcomes of interest (Box 1).

There were no date or language restrictions.

Studies that evaluated interventions, policies, or programs that are enacted at the individual level (e.g., provider or patient level) or organizational level of the health system (e.g., hospital or primary care organization), and do not require change at the level of the national or regional health system were excluded.

**Box 1. Definitions of Included Hypertension Outcomes**

(1) HT awareness. Defined as persons with clinically measured HT who have been diagnosed by a health care professional as hypertensive.

(2) HT treatment. Defined as the use of at least one antihypertensive medication in an individual with known HT.

(3) Antihypertensive medication adherence. Defined as consistently taking the antihypertensive medication regimen as prescribed by the health care provider.

(4) HT control: defined as the achievement of BP below 140/90 mmHg (or other explicitly defined threshold) in individuals being treated for HT, or, alternatively, measured by the mean BP amongst individuals with HT.

### Search Strategy

The search strategy and terms were developed collaboratively with an information specialist. Key words (MeSH terms) and free text terms were identified for each domain of our health systems framework and combined with search terms for HT outcomes to generate the search strategy for the electronic databases Medline, Embase, and Global Health (Text S2). To improve the likelihood of identifying studies from LMICs, modified searches were performed on the following databases: Latin American and Caribbean Health Sciences Literature (LILACS), Africa-Wide Information, Index Medicus for the South-East Asian Region (IMSEAR), Index Medicus for the Eastern Mediterranean Region (IMEMR) Western Pacific Rim Region Index Medicus (WPRIM). All databases were searched from inception to the present day on 8th May 2013. To identify further relevant studies, reference lists of included articles were hand searched and a forward citation search was performed on included studies using Web of Science.

### Study Selection

Two reviewers independently screened the search results by title and abstract for potential eligibility. Full texts of potentially suitable articles were obtained and were further screened for inclusion by two reviewers. Disagreements in the screening of full texts were resolved by a third reviewer with expertise in health systems and this was required for four of the 122 screened papers.

### Data Extraction for Study Setting, Methodology, and Findings

A data extraction form was developed in Microsoft Excel. Data were extracted from each study on study design, setting, health system domains investigated, study methods, and outcomes (Table S1). Where multiple analytical models were used for HT outcomes in a study, data were taken from the analytical model that had the highest level of control for other confounding factors. Data extraction was performed independently by two reviewers and compared and checked for disparities. Erroneous or inconsistent data were identified in one of the included papers, and we attempted to contact the authors of this paper for clarification. Clarification of these data was not forthcoming, so these data were excluded from the analysis.

### Risk of Bias Assessment

Included studies were independently assessed for risk of bias by two reviewers. For observational study designs, risk of bias was assessed using a simple proforma for three domains: selection bias, information bias (differential misclassification and non-differential misclassification), and confounding (Text S3). Assessment of non-differential misclassification took into consideration the reliability of the measure used to report HT outcomes, which was particularly important for medication adherence, where a variety of methods were used for measurement. Risk of bias for each domain was assessed as either low, unclear, or high. Studies that had a low risk of bias in each domain, including a low risk of confounding, were classified as having a low overall risk of bias. For randomized studies the Cochrane risk of bias tool was used [17]. Qualitative studies were evaluated for quality using an adapted version of a checklist used in a previous series of mixed methods systematic reviews incorporating both quantitative and qualitative studies (Text S4) [18],[19].

### Assessment of Context and Complexity Considerations

Due to the recognized importance of context and complexity to health systems research [20], we examined the extent to which included studies describe and explore these factors. We assessed to what extent studies had described the sociodemographic, political, or economic context in which they were conducted and the wider health system setting. We also assessed whether studies demonstrated a consideration of the complexity of health systems, including addressing inter-relationships between different health systems domains, for example, those between financing arrangements and retention of skilled health care workers, as well as interactions with contextual factors, such as the level of poverty or literacy amongst the population being served. This process was performed by one reviewer and checked for consistency by a second reviewer.

### Data Synthesis and Analysis

A narrative synthesis was performed, with studies categorized according to the health system domain they investigated and the setting in which the study was performed. For making causal inferences about reported associations between health systems arrangements and HT outcomes, randomized controlled trials (RCTs) were considered the strongest study design, followed by cohort studies and then case-control studies. Cross-sectional studies and ecological studies, alone, were not considered appropriate for causal inference. Meta-analysis was not conducted as we judged that the included studies were heterogeneous in important aspects, including: populations (different ages and settings), study designs (cross-sectional, case-control, cohort), variable definitions (including different definitions of exposures and outcomes), comparisons (e.g., different type of insurance schemes), and analytical strategies (adjustment for different confounders).

## Results

**Figure 2:**
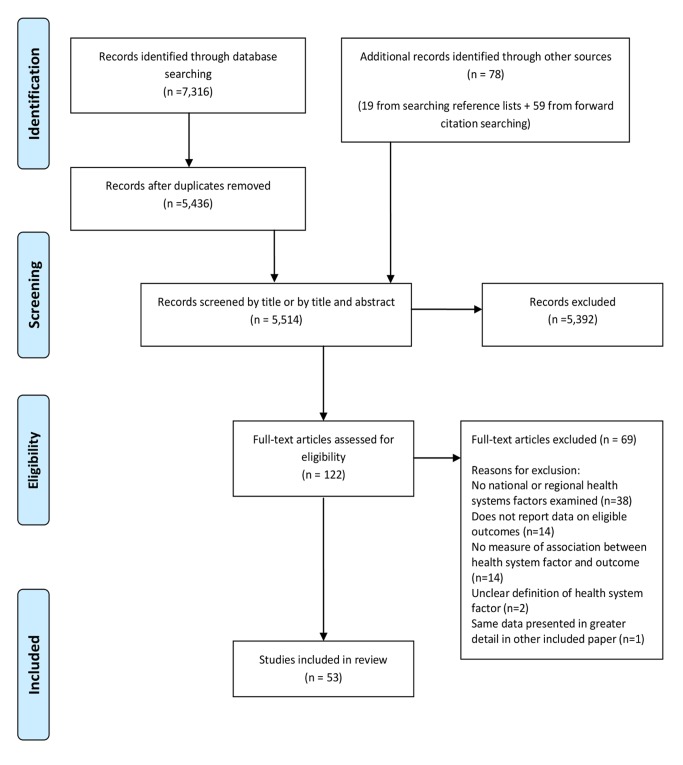
PRISMA flowchart.

The screening process is described using an adapted Preferred Reporting Items for Systematic Reviews and Meta-Analyses (PRISMA) flowchart (Figure 2) [21]. 5,514 articles were screened by title and abstract for inclusion. The full text of 122 of the 5,514 articles was obtained and assessed for eligibility. 53 studies met eligibility criteria for this review. Full details of the included studies, including study design, setting, key findings, and risk of bias assessment can be found in Table S1. 51 of the included studies were quantitative and two were qualitative [22],[23]. Of the 51 quantitative studies, one was a RCT [24]; 12 were cohort studies [25]–[34], two of which were retrospective [30],[34]; three were case-control studies [35]–[37]; 32 were cross-sectional studies; and three were ecological studies [38]–[40]. 42 of the 53 studies (79%) were carried out in countries classified by the World Bank as high-income countries, 36 of which were in the US. Six studies were carried out in upper middle-income countries [38],[41]–[45], three in lower middle-income countries [23],[28],[46], and one in a low-income country [47]. Table 1 describes the health systems factors investigated, classified into the domains of the conceptual framework (Figure 1).

**Table 1:** Health system arrangements investigated by included quantitative studies, classified by health system domain.

Health System Framework Domain^a^	Health System Factor Being Investigated	Number of Studies	Number of Studies and Study Designs	Setting of Studies (Countries)
Physical resources	Distance to health facilities	1	Cross-sectional (1)	Ethiopia (1)
	All physical resources studies	1	Cross-sectional (1)	Ethiopia (1)
Human resources	Level of training/specialism of treating physician	2	Cross-sectional (2)	US (1), Mexico (1)
	Supply of health professionals	2	Cross-sectional (1)	Mexico (1)
	All human resources studies	3	Cross-sectional (3)	US (1), Mexico (2)
Intellectual resources	All intellectual resources studies	0	0 studies	n/a
Social resources	All social resources studies	0	0 studies	n/a
Health system financing	Health insurance status	21	Cohort (2)Case-control (3)Cross-sectional (16)	US (20), Mexico (1)
	Medication costs or medication co-payments	14	Cohort (7)Case-control (1)Cross-sectional (6)	US (9), Finland (1), Brazil (1), Israel (1), China (1), Cameroon (1)
	Co-payments for medical care	3	RCT (1)Cohort (1)Case-control (1)	US (2), Hong-Kong (1)
	Physician remuneration model	2	Cross-sectional (1)Ecological (1)	US (1), Canada (1)
	All financing studies	38	1 RCT (1)Cohort (10)Case-control (3)Cross-sectional (23)Ecological (1)	US (30), Canada (1), Mexico (1), Hong-Kong (1), Israel (1), Finland (1), Brazil (1), China (1), Cameroon (1)
Governance and delivery	Care delivered by private or public provider	3	Cross-sectional (3)	US (1), Greece (1), South Africa (1)
	Routine place of care	6	Cross-sectional (6)	US (6)
	Routine treating physician	7	Case-control (1)Cross-sectional (6)	US (7)
	Either a routine physician or place of care	1	Case-control (1)	US
	All governance and delivery studies	16	Case-control (2)Cross-sectional (14)	US (14), Greece (1), South Africa (1)

a Some studies separately assess more than one health system arrangement.

### Effect of Health System Arrangements on Hypertension Outcomes

#### Physical resources

One study examined the effect of health system factors relating to physical resources (Table 2). This study was conducted in a low-income country, Ethiopia, and examined the effect of distance that patients were required to travel to health facilities providing HT care [47]. The study was cross-sectional in design and had a low risk of bias for all methodological domains assessed. The study reported a moderate positive association between a shorter distance of travel to a health facility and antihypertensive medication adherence (odds ratio [OR] for medication adherence in those with a travel time to health facilities of less than 30 min versus travel time of more than 30 min, 2.02, 95% CI 1.19–3.43).

**Table 2:** Summary of findings of studies examining the associations of arrangements relating to human or physical resources with hypertension outcomes.

Health System Arrangement	Study	Setting and Sample Size	Study Design	Findings (95% CIs Given in Brackets Where Available). ORs Are Adjusted for Confounding Unless Stated Otherwise.	Risk of Bias Assessment
**Physical resources**					
Distance to health facility	Ambaw et al. 2012 [47]	Ethiopia - University hospital, mixed rural and urban population*n* = 384	Cross-sectional	OR for medication adherence travel time to health facilities <30 min versus >30 min 2.02 (1.19–3.43)	Low risk of bias.
**Human resources**					
Grade of treating physician	Federman et al. 2005 [48]	US - All male Veterans Affairs population*n* = 15,893	Cross-sectional	OR for BP control (baseline = 1 for resident). Mid level doctor 1.12 (0.98–1.28), attending 1.23 (1.08–1.39)	Unclear risk of non-differential misclassification.
Physician specialism	Mejia-Rodriguez et al. 2009 [43]	Mexico - Regional Family medicine units*n* = 4,040	Cross-sectional	OR for uncontrolled HT in those treated by non-specialists versus specialists 1.43 (1.20–1.71)	Unclear risk of non-differential misclassification.
Per capita supply of health professionals	Bleich et al. 2007 [41]	Mexico - Nationally representative sample*n* = 2,130	Cross-sectional	OR for HT treatment 1.04 (0.85 to 1.26) and control 0.81 (0.61–1.09) in areas with high versus low supply of health professionals.	Unclear risk of non-differential misclassification.

#### Human resources

Three studies examined the effect of health system factors relating to human resources, none of which had a low risk of bias (Table 2). Two of the three were conducted in an upper-middle income country (both in Mexico), and one was conducted in a high-income country (US).

One US cross-sectional study evaluated the effect of the treating physician's seniority on HT control [48]. This study found a small positive association between seniority of treating physician and HT control. The adjusted OR for HT control was 1.23 (95% CI 1.08–1.39) for patients treated by an attending level physician compared to those treated by a resident level physician.

One Mexican cross-sectional study evaluated the impact of being treated by a specialist on HT control [43]. This study found a moderately increased risk of uncontrolled HT in hypertensive individuals treated by non-specialist physicians (general practitioners) compared to those treated by specialists (adjusted OR 1.43, 95% CI 1.20–1.71).

Another Mexican cross-sectional study evaluated the effect of the density of health professionals and did not find an association with HT treatment or control [41].

#### Intellectual resources

None of the included studies evaluated the effects of health system factors relating to intellectual resources on HT outcomes.

#### Social resources

None of the included studies evaluated the effects of health system factors relating to social resources.

#### Health Systems Financing

38 quantitative studies analyzed the effects of health systems financing on HT outcomes (34 of these studies were conducted in high-income countries and four in middle-income countries). Four different health system arrangements were analyzed, with 21 studies assessing effects of health insurance coverage, 11 examining the effects of medication co-payments or costs, three analyzing co-payments for medical care, and two looking at physician remuneration models.

Twenty of 21 studies evaluating health insurance coverage were conducted in the US and one in Mexico (Table 3). Seven of the 21 studies had a low risk of bias. Two were cohort studies, three were case-control studies, and 16 were cross-sectional studies. 19 of the 21 studies evaluating health insurance reported direct comparisons of HT outcomes in insured and uninsured patients, while two studies only compared private and public insurance schemes. Two cohort studies, both set in the US, compared uninsured patients with insured patients [26],[49]. One of the cohort studies had a 9-y follow-up and found that being uninsured was associated with an increased risk of both unawareness of HT (relative risk [RR] of unawareness in uninsured versus insured patients 1.12, 95% CI 1.00–1.25) and inadequate control of HT (RR of inadequate control in uninsured versus insured patients 1.23, 95% CI 1.08–1.39) [49]. The other cohort study had a follow up period of 30 mo and found that medication adherence was lower in uninsured patients compared to insured patients (OR for medication adherence for uninsured versus insured 0.426, 95% CI 0.282–0.757) [26]. One of two US set case-control studies comparing HT outcomes in uninsured and insured patients reported that insurance was associated with an increased likelihood of HT control (OR for HT control in insured versus uninsured 2.15, 95% CI 1.02–4.52) [36]. The other case-control study reported a non-significant association between being uninsured and having severe uncontrolled HT (OR for severe uncontrolled HT in uninsured versus insured 1.9, 95% CI 0.8–4.6). [35]. Fifteen cross-sectional studies reported comparisons of HT outcomes in insured and uninsured patients. Eight of these 15 studies reported that insurance was associated with improved HT treatment, control or medication adherence [41],[50]–[56]. The seven other cross-sectional studies that compared HT outcomes in insured patients and uninsured patients, reported no significant negative or positive associations between insurance status and HT outcomes [57]–[63]. Two further studies looking at health insurance status compared HT outcomes in patients with public and private health insurance. A case-control study set in the US found increased odds of HT control in patients with private insurance compared to patients with public insurance (OR for HT control 3.40, 95% CI 1.25–9,28) [37]. A cross-sectional study, also set in the US, found no significant association between private or public insurance and HT awareness or treatment, but did report significantly lower levels of systolic blood pressure (BP) in patients with private insurance compared to public insurance (*p*<0.05) [64].

**Table 3:** Findings of quantitative studies examining the association of health insurance status with hypertension outcomes.

Study	Setting and Sample Size	Study Design and Length of Follow-up Where Applicable	Findings (95% CIs Given in Brackets Where Available). ORs Are Adjusted for Confounding Unless Stated Otherwise.	Risk of Bias Assessment
Fowler-Brown et al. 2007 [49]	US. General population of four US communities.*n* = 15,972	Cohort (9-y follow-up)	RR of being unaware of HT 1.12 (1.00–1.25) for uninsured versus insured. RR for inadequate HT control 1.23 (1.08–1.39) for uninsured versus insured.	Unclear risk of sample bias.
Gai and Gu 2009 [26]	US. Nationally representative sample*n* = 3,679	Cohort (30-mo follow-up)	OR of medication adherence: multiple insurance coverage gaps 0.636 (0.418–0.969), uninsured 0.426 (0.282–0.757) versus insured with no coverage gaps (baseline OR = 1)	Unclear risk of differential misclassification bias.
Ahluwalia et al. 1997 [36]	US. Urban, low-income, African-Americans*n* = 221	Case-control	OR of HT control: medical insurance versus no medical insurance 2.15 (1.02–4.52)	High risk of sample bias. Unclear risk of non-differential misclassification bias.
DeVore et al. 2010 [37]	US. Diverse inner-city population attending tertiary cardiology clinic*n* = 154	Case-control	OR of HT control for private versus public insurance = 3.40 (1.25–9.28)	Low risk of bias.
Shea et al. 1992a [35]	US. Hospital-based African American and Hispanic inner-city population*n* = 207	Case-control	OR for severe uncontrolled HT for uninsured versus insured 1.9 (0.8–4.6)	Unclear risk of non-differential and differential misclassification bias.
Angell et al. 2008 [56]	US. Urban NYC population*n* = 1,975	Cross-sectional	Percentage aware of HT with private insurance (baseline) 86.5% (80.3–90.9), Medicare 85.9% (72.8–93.2; *p*>0.05), other government insurance 86.9% (77.3–92.8; *p*>0.05), uninsured 60.2% (46.0–72.8; *p*<0.05)Percentage treated for HT with private insurance (baseline) 76.6% (68.9–82.8), Medicare 81.2% (72.8–93.2; *p*>0.05), other government insurance 74.5% (64.0–82.8; *p*>0.05), uninsured 42.6% (28.7–57.7; *p*<0.05)OR of HT control with Medicare 0.92 (0.36–2.33), other government 0.72 (0.30–1.76), uninsured 0.89 (0.30–2.59) versus private insurance (baseline OR = 1)	Low risk of bias and confounding.
Bautista et al. 2008 [54]	US. Nationally representative sample*n* = 6,100	Cross-sectional	OR of medication non-persistence (non-adherence) with no health insurance 1.88 (1.24–2.83) versus health insurance	Unclear risk of non-differential misclassification bias.
Benkert et al. 2001 [76]	US. Urban Midwest population at nurse-managed center.*n* = 52	Cross-sectional	Mean BP of those uninsured lower than those uninsured (*p*<0.05 for diastolic BP, *p*>0.05 systolic BP).	High risk of sample bias. Unclear risk of non-differential misclassification bias. High risk of confounding.
Bleich et al. 2007 [41]	Mexico. Nationally representative sample*n* = 2,130	Cross-sectional	OR for HT control with seguro popular (insured) versus uninsured, for treatment 1.50 (1.27–1.78), and for control 1.35 (1.00–1.82)	Unclear risk of non-differential misclassification bias.
Brooks et al. 2010 [50]	US. Framingham cohort*n* = 1,384	Cross-sectional	Men and women treated less when uninsured (OR 0.19 [0.07–0.56] and 0.31 [0.12–0.79], respectively). Men less controlled when uninsured (OR 0.17 [0.04–0.68]), not women.	Low risk of bias.
Duru et al. 2007 [51]	US. Nationally representative sample*n* = 3,496	Cross-sectional	OR for HT control (ref 1.0 for private insurance), Medicare = 0.80 (0.61–1.05), Medicaid 0.75 (0.47–1.20), no insurance 0.63 (0.44–0.92).	Low risk of bias.
Ford et al. 1998 [63]	US. Nationally representative sample*n* = 1,724	Cross-sectional	Found no differences in HT awareness, treatment, or control with no health insurance, Medicaid only, or other health insurance compared to those insured fully.	High risk of non-differential misclassification bias.
He et al. 2002 [53]	US. General population*n* = 4,144	Cross-sectional	OR of HT control with government insurance = 1.08 (0.70–1.68); private insurance = 1.59 (1.02–2.49), versus no insurance.	Low risk of bias.
Hill et al. 2002 [57]	US. Inner-city African American men presenting to the emergency department*n* = 309	Cross-sectional	No significant association between health insurance status and HT control.	Unclear risk of sample bias.
Hyman and Pavlik 2001 [58]	US. Nationally representative sample*n* = 10,576	Cross-sectional	OR for uncontrolled HT with insurance versus without 1.30 (0.79–2.13)	Low risk of bias.
Kang et al. 2006 [59]	US. Low SES Korean-American elderly*n* = 146	Cross-sectional	OR of HT treatment with any insurance 2.41 (0.91–6.39), Medicare 2.06 (0.66–6.42), Medicaid 3.21 (0.89–11.61), private insurance1.46 (0.29–7.39) versus none. No association between insurance type and control.	High risk of sample bias. Unclear risk of non-differential misclassification bias.High risk of confounding
Moy et al. 1995 [55]	US. Nationally representative sample*n* = 6,158	Cross-sectional	OR of non-treatment of HT with Medicare or Medicaid versus private 1.19 (0.99–1.41), Uninsured versus private 1.49 (1.18–1.89)	High risk of non-differential misclassification bias. Unclear risk of differential misclassification bias.
Nguyen et al. 2011 [71]	US. Population sample from NYC*n* = 1,334	Cross-sectional	Public versus private insurance. OR for HT awareness 1.2 (0.4–4.1), treatment 1.1 (0.4–3.6). Average SBP lower with private insurance versus public.	Low risk of bias.
Shea et al. 1992b [60]	US. Hospital-based African American and Hispanic inner-city population*n* = 207	Cross-sectional	Health insurance was not significantly associated with medication adherence in a multivariable model.	High risk of sample bias. Unclear risk of non-differential misclassification bias.
Turner et al. 2009 [62]	US. Mostly African American women in Philadelphia*n* = 300	Cross-sectional	OR: In the past year had to go without usual BP medications because not covered (yes) 1.29 (0.26–9.49) versus no	High risk of sample bias.
Wyatt et al. 2008 [61]	US. African American population from Jackson, MS*n* = 4,986	Cross-sectional	No association reported between health insurance status and HT awareness, treatment, or control.	Unclear risk of sample bias.

RR, risk ratio; SES, socioeconomic status.

Fourteen quantitative studies measured the association of medication co-payments or costs with HT control or treatment adherence, nine of which were set in the US, and one in each of Cameroon, China, Finland, Israel, and Brazil (Table 4). Two of the 14 studies had a low risk of bias. Seven of the 14 studies were cohort studies, one was a case-control study, and six were cross-sectional studies. All seven cohort studies reported associations between increased medication costs or co-payments and reductions in HT control or reduced adherence to antihypertensive medication [25],[27],[29],[30],[32],[34],[65], although for one of these seven cohort studies, the association between increased co-payments and reduced medication adherence was only found for low medication co-payments, and at high co-payment levels medication adherence was actually found to increase (OR for medication adherence versus baseline of 1 for US$0 co-payments was 0.72 for US$1–US$9 co-payments (*p*<0.05), 1.02 for US$10–US$29 co-payments (*p*>0.05), and 1.32 for co-payments >US$30 (*p*<0.05)) [34]. Five cross-sectional studies and one case-control study also examined associations between medication co-payments or costs and HT control or adherence to antihypertensive medication [42],[45],[66]–[68]. All six of these studies reported significant associations between reduced co-payments or costs and improved HT control or medication adherence. One of these cross-sectional studies, set in China, also looked at the effect of medication costs on HT treatment rates. This study found that 0.0% of people given access to free antihypertensive medication remained untreated compared to 14.7% who had to pay for medication (*p*<0.001) [45]. One cross-sectional study set in Cameroon examined the association of medication costs with HT awareness and did not find one, although the confidence intervals were wide (OR for HT awareness for high medication cost versus low medication cost 0.44, 95% CI 0.07–2.75) [46]. Two qualitative studies, one from the US and one from Nigeria, cited cost of medications as a barrier to medication adherence [22],[23].

**Table 4:** Findings of quantitative studies examining the association of medication or medical care costs or co-payments with hypertension outcomes.

Study	Setting and Sample Size	Study Design	Findings (95% CIs Given in Brackets Where Available). ORs Are Adjusted for Confounding Unless Stated Otherwise.	Risk of Bias Assessment
**Medication costs and co-payments**				
Briesacher et al. 2009 [34]	US. Nationally representative sample of adults in employment.*n* = 125,397	Cohort (12-mo follow-up)	OR for medication adherence versus baseline of 1 for US$0 co-payments. OR = 0.72 (*p*<0.05) for US$1–US$9 co-payments, OR = 1.02 (p>0.05) for US$10–US$29 co-payments, OR = 1.32 (*p*<0.05) for co-payments >US$30	Unclear risk of sampling bias
Elhayany and Vinker 2001 [25]	Israel. Mixed Arab/Jewish patients from Ramle and Lod (deprived populations)*n* = 260	Cohort - before and after study of intervention. (2-y follow-up)	Systolic BP and diastolic BP reduced by 8 and 3.2 mmHg, respectively, 24 mo following intervention to eliminate prescription co-payments (*p*<0.001).	High risk of selection bias. High risk of confounding.
Hsu et al. 2006 [27]	US. Sample from Kaiser Permanente HMO in Northern California*n* = 104,948	Cohort (12-mo follow-up)	OR for poor HT control = 1.05 (1.00–1.09) in capped versus uncapped drug benefits	Low risk of bias.
Li et al. 2012 [29]	US. Nationally representative sample. Looking at effect of the Medicare Part D medication coverage gap on medication adherence*n* = 54,594	Cohort (Length of follow-up unclear)	In 2006 Medicare Part D had a gap in coveragefor prescription payments, where recipients had to cover 100% of drug costs above a threshold of US$2250 per annum. Some insurance plans covered this gap in coverage. ORs for non-adherence versus a control group of people entitled to complete low income medication subsidy were as follows: brand name and generic gap coverage 1.00 (0.88–1.15), generic only gap coverage 1.50 (1.30–1.73), no gap coverage 1.60 (1.50–1.71).	Unclear risk of selection bias and non-differential misclassification bias.
Maciejewski et al. 2010 [30]	US. Veterans Affairs Medical Centers*n* = 7,090	Cohort (34-mo follow-up)	2 y after co-payment increase: difference in adherence = −3.2% (−3.1 to −3.3) in co-payers compared to exempt controls.	Low risk of bias.
Pesa et al. 2012 [32]	US. Nationally representative sample*n* = 26,688	Cohort (12-mo follow-up)	For every US$1.00 increase in cost sharing, PDC decreased by 1.1 d (*p*<0.0001)	Unclear risk of non-differential misclassification bias.
Schoen et al. 2001 [65]	US. Uninsured patients at an inner-city university-based outpatient clinic*n* = 137	Cohort (2-y follow-up)	Percent people with uncontrolled HT reaching therapeutic goal increased from 19.0% at baseline to 36.8% at 6 mo (*p*<0.001) and 65.8% at 24 mo (*p*<0.01) after intervention to increase access to free medications.	High risk of selection bias. Unclear risk of non-differential misclassification bias. High risk of confounding.
Ahluwalia et al. 1997 [36]	US. Low-income, African-Americans in an urban ambulatory hospital*n* = 221	Case-control	OR of HT control when cost not a deterrent to purchasing medications) versus cost is a deterrent 3.63 (1.59–8.28)	Unclear risk of differential misclassification bias.
Gandelman et al. 2004 [68]	US. General sample of University Medical Center patients Westchester, NY*n* = 614	Cross-sectional	38% of self-pay or Medicare patients (co-payers) have controlled BP versus 70% of Medicaid/privately insured (no co-payments) *p*<0.001.	High risk of confounding.
Jokisalo et al. 2002 [67]	Finland. Nationally representative sample*n* = 1,561	Cross-sectional	Medication adherence increases with presence of special reimbursement payments for medication costs (*p*<0.001 between groups)	High risk of selection bias and confounding. Unclear risk of non-differential misclassification bias.
Mbouemboue et al. 2012 [46]	Cameroon. Mixed rural and urban sample in Adamawa Region*n* = 117	Cross-sectional	OR for HT awareness (baseline 1 for low cost of medications): medium cost 0.35 (0.06–2.07), high cost 0.44 (0.07–2.75)	Unclear risk of non-differential misclassification bias.
de Santa-Helena et al. 2010 [42]	Brazil. Patients from family health units in Blumenau*n* = 595	Cross-sectional	OR for non-adherence: Those who pay for medications versus those who have drugs provided by SUS (health service) = 4.9 (1.6–15.3)	Unclear risk of non-differential misclassification bias.
Yoon and Etner 2009 [66]	US. Generally representative US sample, all insured.*n* = 83,893	Cross-sectional	Amongst people with low to median baseline levels of adherence to medication (10th, 25th, and 50th centile) increased co-payments, at all levels, had a significant negative effect on adherence to antihypertensive medication. (see Table S1 for detail).	Unclear risk of confounding.
Yu et al. 2013 [45]	China. Low income rural residents in Shandong province.*n* = 204	Cross-sectional with matched control group	0% of intervention group (free-medication) untreated for HT compared to 14.7% in control (pay for medication) group (*p*<0.001)Significantly improved adherence to medication in intervention group compared to control group (*p* = 0.034)12.7% in intervention group versus 11.8% in control group have controlled HT. *P* = 0.831	High risk of confoundingUnclear risk of non-differential misclassification bias.
**Co-payments for medical care**				
Keeler et al. 1985 [24]	US. Nationally representative sample, subset of RAND study 3,958	RCT (3–5-y follow-up)	Mean difference in diastolic BP (free plan - cost sharing plans) = −1.9 mmHg (−3.5 to −0.3) *p*<0.05.Mean difference in systolic BP hypertensive patients = −1.8 mmHg (−4.5 to 0.6) *p*>0.05.	High risk of participant and personnel blinding. Unclear risk of random sequence generation, allocation concealment, and blinding of outcome assessment. Low risk of selective reporting and incomplete outcome data.
Wong et al. 2010 [33]	Hong Kong. Chinese patients in primary care*n* = 83,884	Cohort (unclear length of follow-up)	OR for medication adherence fee payers versus fee waivers 1.14 (1.09–1.19)	Low risk of bias.
Ahluwalia et al. 1997 [36]	US. Low-income, African-Americans in an urban ambulatory hospital*n* = 733	Case-control	OR of control when cost of care not a deterrent versus cost as a deterrent 2.35 (1.19–4.67)	Unclear risk of differential misclassification bias.

PDC, proportion of days covered by medication.

Three studies assessed co-payments or costs of medical care (not simply medications), two of which were conducted in the US (an RCT and a case-control study) and one in Hong Kong (a cohort study). (Table 4) [24],[33],[36]. One of the three studies, the cohort study from Hong-Kong, had a low risk of bias [33]. The RCT reported a higher mean BP level amongst individuals with HT who had cost-sharing insurance plans compared to those with free care, although this was non-significant for systolic BP [24]. The adjusted mean difference in diastolic BP between the two groups was 1.9 mm mercury (mmHg) (95% CI 0.3–3.5 mmHg) and the adjusted mean difference in systolic BP was 1.8 mmHg (95% CI −0.6 to 4.5 mmHg). The case-control study reported that cost of care was a deterrent to BP control (adjusted OR for BP control when cost not a deterrent versus cost as a deterrent: 2.35, 95% CI 1.19–4.67) [36]. The cohort study, which was set in Hong Kong, found, conversely, that being a fee payer was associated with improved adherence to prescribed antihypertensive medications compared to fee waivers (adjusted OR for adherence fee payers versus fee waivers 1.14, 95% CI 1.09–1.19) [33].

Two studies evaluated the association of physician remuneration models with HT control or treatment adherence, one an ecological study set in Canada, and one a US cross-sectional study (Table 5). Neither study had a low risk of bias. The US study reported improved rates of HT control amongst patients treated under a capitation model compared to fee-for service patients (adjusted OR for HT control 1.82, 95% CI 1.02–3.27 for capitation versus fee-for-service patients) [69]. The Canadian study reported highest rates of HT treatment and control among practices using a capitation model, compared to fee-for-service and salary models [40]. HT awareness levels were highest in practices with a fixed salary remuneration model.

**Table 5:** Findings of quantitative studies examining the association of physician remuneration models with hypertension outcomes.

Study	Setting and Sample Size	Study Design and Length of Follow-up Where Applicable	Findings (95% CIs Given in Brackets Where Available). ORs Are Adjusted For Confounding Unless Stated Otherwise.	Risk of Bias Assessment
Tu et al. 2009 [40]	Canada. Primary care in Ontario.*n* = 135	Ecological	Differences in rates of HT awareness (*p* = 0.22), treatment (*p* = 0.01) and control (*p*<0.01) between capitation, salary, and fee for service practices. Highest rates of awareness in salary practices. Highest rates of treatment and control in capitation practices.	Unclear risk of selection bias.
Udvarhelyi et al. 1991 [69]	US. Health care facilities with both capitation and fee-for service patients*n* = 246	Cross-sectional	OR for HT control = 1.82 (1.02–3.27) for HMO (capitation) versus fee-for-service patients.	Unclear risk of selection bias. Unclear risk of misclassification bias.

#### Delivery and Governance

16 studies examined the effects of health systems arrangements relating to delivery and governance on HT outcomes (Table 6). Fifteen of these studies were conducted in high-income countries and one in a LMIC. Four different health systems arrangements were analyzed, with six studies evaluating having a routine place of care for HT management, seven studies evaluating having a routine physician for HT care, one study evaluating having either a routine place or physician for HT care, and four studies assessing whether care was delivered by the private versus the public sector.

**Table 6:** Findings of studies examining health systems arrangements relating to health systems delivery and governance.

Study	Setting and Sample Size	Study Design	Findings (95% CIs Given in Brackets Where Available)ORs Are Adjusted for Confounding Unless Stated Otherwise.	Risk of Bias Assessment
**Routine place of care for HT**				
Angell et al. 2008 [56]	US. Urban population from NYC*n* = 1,975	Cross-sectional	HT awareness with a routine place of care 85.1% versus 65.5% without (*p*<0.05). HT treatment with routine place of care 76.4% versus without 42.1% (*p*<0.05). OR for HT control without a routine place of care 0.21 (0.07–0.66) versus with a routine place of care	Low risk of bias.
He et al. 2002 [53]	US. General population*n* = 4,144	Cross-sectional	OR for control for same health facility of care 2.77 (1.88–4.09) versus lack of same facility of care	Low risk of bias.
Hyman and Pavlik, 2001 [58]	US. Nationally representative sample*n* = 10,576	Cross-sectional	OR for lack of awareness of HT: has usual source of care: 1.12 (0.87–1.43) versus has no usual source of care. OR for acknowledged uncontrolled HT: has usual source of care: 1.07 (0.63–1.84) versus no usual source of care.	Low risk of bias.
Moy et al. 1995 [55]	US. Nationally representative sample*n* = 6,158	Cross-sectional	OR for no HT treatment (reference 1 for physician's office) Clinic OR = 1.07 (0.90–1.28), Emergency department OR = 1.36 (0.73–2.55), No usual place of care OR = 3.94 (3.05–5.08)	High risk of non-differential misclassification. Unclear risk of differential misclassification.
Nguyen et al. 2011 [71]	US. Population sample from NYC*n* = 1,334	Cross-sectional	HT awareness: OR = 1.0 (0.2–5.6) no usual care versus usual place of care (baseline). HT treatment OR = 0.2 (0.1–0.8) no usual care versus usual place of care (baseline). Systolic BP 16.4 mmHg higher with no usual place of care (*p* = 0.02).	Low risk of bias.
Spatz et al. 2010 [70]	US. Nationally representative sample*n* = 6,672	Cross-sectional	APR for being untreated = 2.43 (1.88–2.85) for no usual source of care versus having a usual source of care.	Low risk of bias.
**Routine physician for HT care**				
Shea et al. 1992a [35]	US. Hospital-based African American and Hispanic inner-city population in NYC*n* = 207	Case-control	OR for severe uncontrolled HT with no routine physician 3.5 (1.6–7.7) versus with a routine physician	Unclear risk of differential and non-differential misclassification.
Ahluwalia et al. 2010 [73]	US. West Virginian women in a screening initiative*n* = 733	Cross-sectional	OR of having uncontrolled HT with a regular physician 0.34 (0.13–0.88) versus no regular physician	High risk of sample bias. Unclear risk of non-differential misclassification bias.
He et al. 2002 [53]	US. General population*n* = 4,144	Cross-sectional	OR for HT control same health provider of care 2.29 (1.74–3.02) versus lack of same provider of care	Low risk of bias.
Hill et al. 2002 [57]	US. Inner-city African American men presenting to the emergency department*n* = 309	Cross-sectional	Non-significant association between regular MD for HT care and HT control, magnitude of association not reported in paper.	Unclear risk of sample bias.
Moy et al. 1995 [55]	US. Nationally representative sample*n* = 6,158	Cross-sectional	OR for no treatment (reference 1 for general or family practitioner), Internist OR = 0.82 (0.67–1.00), Non primary care physician OR = 1.20 (0.97–1.49), No particular physician OR = 2.61 (2.15–3.18)	High risk of non-differential misclassification. Unclear risk of differential misclassification.
Shea et al. 1992b [60]	US. Hospital-based African American and Hispanic inner-city population*n* = 207	Cross-sectional	OR for non-adherence for lack of primary care physician 2.9 (1.36–6.02 versus presence of primary care physician.	High risk of sample bias. Unclear risk of non-differential misclassification bias.
Victor et al. 2008 [72]	US. Mostly non-Hispanic African Americans from Dallas County*n* = 1514	Cross-sectional	OR for HT awareness 3.81 (2.86–5.07), treatment 8.36 (5.95–11.74), and control 5.23 (3.30–8.29): Has a regular physician versus has no regular physician.	Low risk of bias.
**Routine physician or place of care for HT**				
Ahluwalia et al. 1997 [36]	US. Low-income, African-Americans in an urban ambulatory hospital*n* = 221	Case-control	OR of HT control: Regular source of care 7.93 (3.86–16.29) versus no regular source of care.	Unclear risk of differential misclassification.
**Private versus public provision of care**				
Dennison et al. 2007 [44]	South Africa. Peri-urban black South Africans*n* = 403	Cross-sectional	No significant effect of provider type on systolic BP or odds of BP control below threshold (>140 mmHg systolic and >90 mmHg diastolic BP). Diastolic BP 3.29 mmHg greater in public versus private sector (*p* = 0.042).	Unclear risk of sample bias.
Kotchen et al. 1998 [75]	US. Inner-city African American population from Milwaukee*n* = 583	Cross-sectional	Unadjusted OR for HT control: Private provider 1.20 (0.62–2.32) versus non-private provider	High risk of confounding. Unclear risk of sample bias.
de Santa-Helena et al. 2010 [42]	Brazil. Patients from family health units in Blumenau*n* = 595	Cross-sectional	OR for non-adherence: Treated by public health service (SUS) 1.8 (1.1–2.7) versus private medical provider.	Unclear risk of non-differential misclassification.
Yiannakopoulou et al. 2005 [74]	Greece. Patients admitted for elective surgery in Athens.*n* = 1,000	Cross-sectional	Medication adherence with private physician 25.1% versus 10% of those with physician in rural areas and 8.8% of with physician from the National Health System (*p*<0.005 between groups)	High risk of confounding. Unclear risk of non-differential misclassification.

APR, adjusted prevalence ratios.

All six studies analyzing having a routine place of care for HT were conducted in the US, and all were cross sectional in design, with five of the six having a low risk of bias. Five of these six studies reported a significant association between a routine place of care and improved HT awareness, treatment, or control [53],[55],[56],[70],[71]. One study found no association between a routine place of care and HT awareness or control [58]. No studies analyzed medication adherence.

Of the seven studies assessing the effects of having a routine physician for HT care, all were conducted in the US. Two of the seven studies had a low risk of bias. One was a case-control study and six were cross-sectional studies. The case-control study and five of the six cross-sectional studies found that having routine care from the same physician was significantly associated with an improvement in HT awareness, treatment control, or medication adherence [35],[53],[55],[57],[60],[72],[73]. One study did not find a significant association between a routine physician and HT control [57].

A single case-control study, which did not have a low risk of bias, analyzed having either a routine place of care or physician for HT care in the US [36]. It found that having either was strongly associated with improved HT control, with some imprecision in the effect estimate (adjusted OR for HT control 7.93, 95% CI 3.86–16.29 for a regular source of care versus no regular source of care).

Four studies assessed private versus public provision of care, with one set in each of South Africa, US, Brazil, and Greece. All four studies conducted were of cross-sectional design and none had a low overall risk of bias. One study set in Brazil found that non-adherence to antihypertensive medication was more likely in patients treated in the public versus private sector (OR for non-adherence in patients treated by public health service versus private medical provider 1.8, 95% CI 1.1–2.7) [42]. One study, set in the US, found no significant association of provider type with systolic BP or odds of blood pressure control below a threshold of 140 mmHg systolic and 90 mmHg diastolic BP, but did find that diastolic BP was 3.29 mmHg greater in patients treated by the public versus private sector (*p* = 0.042) [44]. The two other studies evaluating public versus private provision had a high risk of confounding, one of which was set in Greece and one in the US. The study set in Greece found increased rates of medication adherence in patients treated by a private physician compared to those treated in the National Health System (medication adherence with private physician 25.1% versus 10% of those with a physician in rural areas and 8.8% of with a physician from the National Health System, *p*<0.005 for between group differences) [74]. The study set in the US did not provide strong evidence of improved HT control in patients cared for by private providers (unadjusted OR for HT control for private versus non-private provider 1.20, 95% CI 0.62–2.32) [75].

### Interventions Involving More Than One Health System Building Block

Four studies were included that evaluated outcomes associated with complex regional or national health policy interventions, which incorporated components from more than one health system domain (Table 7). Two of these studies were conducted in high-income countries, one in Finland and one in Trinidad and Tobago, one was conducted in a higher-middle-income country, Iran, and one was conducted in a lower middle-income country, Cameroon [28],[31],[38],[39]. None of the four studies had a low risk of bias. All four studies showed improvements in HT outcomes after the delivery of the intervention, although for one study there is a high probability that this could be due to random sampling error [38].

**Table 7:** Description and summary of findings of studies evaluating complex national or regional interventions incorporating components from more than one health system building block.

Study, Setting and Sample Size	Study Design	Summary of Intervention	Health System Building Blocks Included	Summary of Findings	Risk of Bias Assessment
Nissinen et al. 1983 [31]North Karelia – Finland*n* = 3,002	Cohort study with control area – 5-y follow-up from 1972–1977	Introduction of systematic HT care within the existing primary health care structure.The program featured public health education, training of health personnel, reorganization of primary care services, and creation of an information system.	1. Human resources2. Physical resources3. Delivery and governance.	BP levels fell further in both hypertensive men and women in intervention region compared to control region (*p*<0.001)	High risk of selection biasHigh risk of confounding.
Labhardt et al. 2010 [28]Central Region, Cameroon.*n* = 493	Cohort study – before and after intervention, no control group.Median follow up 102 d.	Integration of care for HT and type 2 diabetes into the existing primary health care system by task shifting from physicians in hospitals to non-physician clinicians in health centers.The intervention included training, equipment and regional supervision and monitoring. Local treatment protocols were adapted from international guidance.	1. Human resources2. Physical resources3. Intellectual resources4. Delivery and governance.	Fall in BP from baseline to follow up: Systolic BP fell by −26.5 mmHg (95% CI −12.5 to −40.5). Diastolic BP fell by 17.2 mmHg (95% CI −7.1 to −27.3)	High risk of selection bias and differential misclassification bias.
Khosravi et al. 2010 [38].Iran, Intervention areas Ifsahan and Najaf-Abad*n* = 12,514/9,572 (pre-/post- intervention survey)	Ecological study – surveys performed before and after intervention. (6-y follow-up) Reference area included.	Ifsahan Healthy Heart Program:Complex regional intervention incorporating 3 strategies1. Educating health professionals in HT management (includes publication of local guidelines).2. Public education.3. Occasional free BP measurement and cardiovascular risk assessment services.	1. Human resources2. Intellectual resources3. Delivery and governance.	Improvement in BP awareness, treatment and control in intervention area (*p*<0.001 for all outcomes).Improvements also seen in reference area. (*p*<0.05 for all outcomes)	High risk of confounding.
Gulliford et al. 1999 [39].Trinidad and Tobago.*n* = 690/1,597 (pre-/post intervention survey)	Ecological study – surveys performed before and after intervention. (5-y follow-up)	National intervention to improve diabetes care in Trinidad and Tobago. Intervention included:1. Evaluation of diabetes care and feedback of findings.2. Training workshops for doctors.3. Publication and dissemination of guidelines.	1. Human resources2. Intellectual resources3. Delivery and governance.	Adjusted OR for BP control amongst diabetics post intervention versus pre-intervention = 1.24 (95% CI 0.84–1.85)	High risk of selection bias. High risk of non-differential misclassification.

### Considerations of Context

Seventeen of 53 included studies gave no information about the socio-demographic, political, or economic context in which the study was conducted [24],[26],[30]–[32],[39],[42],[47],[54],[55],[58],[63],[67],[69],[70],[74],[75]. Of the 33 studies that provided contextual information, this varied from single phrases or sentences to more detailed contextual information (Box 2). 28 of 53 included studies gave no description of the national or regional health system where the study was conducted [22],[26],[31]–[34],[37]–[39],[43],[46],[53]–[56],[58]–[62],[66]–[68],[72],[74]–[76]. Where a description of the health system was given it was in most cases limited to a brief sentence on insurance coverage or financing arrangements. A minority of studies, usually those carried out in low-income countries, gave more comprehensive descriptions of some aspects of the health system (Box 2).

**Box 2. Examples of Contextual Information and Descriptions of the Health System from Included Studies**

[set in a] “contemporary multi-ethnic urban community” [72], or “developing country setting” [38]

“Among industrialized countries, only the United States lacks universal healthcare.” [63]

“These cities have mixed Arab-Jewish populations and are among the poorest in Israel, defined by the Israeli Social Security Agency as having an SES in the lowest 10% of the population.” [25]

[On a regional health system in Cameroon] “When the program for hypertension and diabetes started in 2007, there were 79 peripheral clinics in the area offering nurse-led primary health care. Four of these had a physician among the staff; the remaining 75 were exclusively led by Non Physician Clinicians. Most (78%) of the peripheral clinics were public where consultations are usually free of charge but diagnostics and drugs for curative services have to be paid out-of-pocket. The area also has eight district hospitals and two missionary hospitals.” [28]

## Considerations of Health Systems Complexity

Eleven of the 53 studies discussed linkages or interdependencies between health system domains [24],[28],[35],[36],[41],[57]–[59],[63],[69],[70]. Eight of these 11 studies discussed the importance of the interdependence between health system financing and structures relating to the delivery of care, with many emphasizing the link between low levels of health insurance coverage in certain US settings and a lack of structures providing access to regular high quality medical care [24],[35],[36],[58],[59],[63],[69],[70]. One study described the link between wider social factors and factors relating to health system financing in creating a barrier to care for African American men in the US: “unemployment and lack of health insurance are highly intercorrelated and constitute apparently insurmountable barriers to traditional medical care for HBP.” [57]. One study, set in Mexico, discussed the positive interaction between the presence of health insurance (financing) and the supply of health professionals (human resources) in improving HT outcomes [41].

Twenty-three of 53 studies considered how HT outcomes may be influenced by relationships between health systems factors and contextual factors, such as socioeconomic status, as well as individual factors, such as gender, age, or co-morbidities [22]–[24],[31],[36],[41],[47],[50],[54]–[57],[59],[63],[66],[70]–[72]. Five of these 23 studies used an established framework, such as the Anderson-Aday model [36],[55], or the Precede-PROCEED model to describe the multiple factors, including health system factors, that might determine HT outcomes at the individual level [44],[57],[59].

## Discussion

Despite the limited scope and variable quality of literature found, as well as the context specificity of the findings, it remains possible to make inferences about the effect of some health system arrangements on HT outcomes. This is particularly the case for the characteristics of the US health system, which while unique among high income countries has features that can be found in other parts of the world. Evidence from longitudinal studies reported here suggests a small positive impact of the presence of health insurance in the US on HT awareness and control, and adherence to antihypertensive medication [26],[49]. This is supported by most, but not all case-control and cross-sectional studies [36],[41],[50]–[56]. However, these findings can be considered in relation to analogous studies, such as a 2008 systematic review of longitudinal studies that was confined to those in the US, which found improved long-term health outcomes, including reduced mortality, in insured patients compared to uninsured patients [77]. We also found an association, in both longitudinal and cross-sectional studies, between reduced co-payments or costs for medications or medical care and improved HT control or treatment adherence in multiple studies in US settings [24],[27],[29],[30],[32],[34],[36],[65],[66],[68], although in one of these studies, by Briesacher et al., the relationship between reduced co-payments and treatment adherence was only found for low levels of medication co-payments, while the highest levels of co-payments (>US$30) were, surprisingly, associated with improved medication adherence. The study authors do not provide an explanation for this result in the paper, but it could be that the subgroup of patients with co-payments of US$30 or more for medications have shared characteristics that were not analyzed in this study, such as high socioeconomic status, which may confound the association between co-payment levels and medication adherence. The association between reduced medication co-payments and improved HT outcomes was replicated in single studies from China, Finland, Israel and Brazil [25],[42],[45],[67] but not in a study of Hong Kong Chinese, which found that fee payers had improved medication adherence compared to those with fee waivers [33]. The finding of an association between reduced medication co-payments and improved HT outcomes is intuitive and suggests that costs of medications or health care consultations may act as a barrier to optimal HT care in the US, and potentially other settings, including LMICs. A relationship between increased medication co-payments and treatment discontinuation has also been reported for diabetes care in the US [78].

Although lacking longitudinal studies, we found a large positive association between having a routine physician or place of care for HT management and treatment, awareness, control, and adherence to antihypertensive treatment, again in the US [35],[36],[53],[55],[56],[60],[70]–[73]. This finding is consistent with a recent systematic review of the effect of a usual source of care, showing an association with improved preventive services and chronic disease control [79]. Although it is unclear whether having a routine physician or a place for HT care is more important, this may matter less than the implication that the absence of a consistent source of care reduces awareness, treatment, and control of HT. It is possible, however, that this effect is linked to health system financing arrangements, as those without insurance or facing high co-payments may be least likely to have consistent access to care [70]. There were no longitudinal studies looking at differences in outcomes of HT management provided by the private or public sector, and the four cross-sectional studies considering this question were all at risk of bias, were in different settings, and had different findings, so general inferences were not possible [42],[44],[75],[80].

All four included studies that evaluated complex multi-component national or regional policy interventions reported some improvement in HT care. These studies had significant methodological flaws including, in some cases, a lack of an adequate control group, precluding attribution of the improvement in HT outcomes to the intervention. However, despite their limitations, these studies may be useful for policymakers seeking to understand ways to strengthen health systems for chronic disease care, particularly in LMICs [6],[15]. Labhardt et al., for example, demonstrated the feasibility of task shifting from physicians to non-physician health care workers for HT management in Cameroon, outlining the integrated interventions across multiple health system domains required to deliver improvements in health outcomes [28].

Research on health systems factors influencing HT care is unequally distributed geographically. There is a lack of evidence from LMICs, which bear around three-quarters of the global HT burden [1]. Furthermore, even in high-income countries, health systems barriers to care have been seen mainly as financial, while the understanding of how a complex mix of other factors influence care is relatively new. Intellectual and social resources, such as the production and use of knowledge, social capital, and systems for communication have only recently emerged as distinct areas of research. As a result we found only a small number of studies examining the impact of health system factors relating to human resources or physical resources, and no studies evaluating the impact of intellectual or social resources. This meant we were unable to make firm conclusions about the effects of these factors on HT outcomes.

A number of included studies used models to conceptualize the mechanisms by which health systems factors may interact with other key variables to influence HT outcomes. For example Moy et al. and Ahluwalia et al. (1997) used the Anderson-Aday model, which illustrates how three types of population characteristics can influence medical care for HT [36],[55]. Factors relating to health systems such as the presence of a usual source of care or health insurance are seen as “enabling” factors for medical care. These “enabling” factors interact with “predisposing” factors such as ethnicity, gender, and socioeconomic status, and “need characteristics” such as health status to determine access and outcomes of medical care. Models such as Anderson-Aday are useful to the extent that they can help demonstrate how health systems factors may interact with other key factors in determining HT outcomes. However, the studies reviewed here lack quantitative and qualitative data on the nature and strength of these interactions, highlighting an important gap to be addressed by future research.

### Study Limitations and Strengths

The majority of included quantitative studies were cross-sectional, and the few longitudinal studies we did find were restricted to either health system arrangements relating to financing or to evaluating the effects of complex multi-component interventions. Inferences about temporal and potentially causal relationships between health systems arrangements and HT outcomes, could, therefore, only be made for a limited number of factors. In addition, included quantitative studies were of variable methodological quality, with only one being randomized and a minority having a low risk of bias for all assessed methodological domains.

When considering the findings of this review, the risk of publication bias cannot be ruled out, particularly for the positive findings relating to health insurance status, medication and treatment costs and co-payments, and presence of a routine setting of care, where it is possible that studies with null findings are under-published. It was not possible to produce an Egger funnel plot to formally assess the risk of publication bias, for the same reasons that meta-analysis was not performed, namely the heterogeneity in the study designs, outcome measures, analysis strategies, and populations in the included studies. Reporting bias within individual studies may also be a factor, many of which might have explored the effects of multiple factors on HT outcomes, and may have failed to report results for health system arrangements which did not show significant effects. The lack of published protocols for the included studies did not allow us to estimate the magnitude of this potential bias. A strength of the review is the addition of forward and backward searching methods to the initial database search for articles. A number of additional studies were identified using these methods, before reaching a saturation point at which the only relevant studies being identified were already included. We included only two qualitative studies, which did not contribute important data about the views of policymakers and health care workers on health systems factors affecting HT care, contrary to what we had initially hoped.

The use of a conceptual health systems framework facilitated the conduct of the review, enabling systematic generation of terms for the search strategy and for classification of included studies according to the domains in the conceptual framework. However, the classification and reporting of our findings according to health system domain does not encourage the integrated view of health systems that the framework promotes. For example, classifying the effect of usual source of care into the domain of health systems governance and delivery obscures the fact that the delivery of care from a regular source is very much dependent on human and physical resources inputs to the health system. The difficulties in presenting such complexities are perhaps a reflection of the fact that few of the included studies explored inter-linkages between health system components, with the majority exploring the association between health system arrangements and HT outcomes as simple linear relationships. There were some notable exceptions, however; one study, for example, examined the interaction of insurance status and the presence of a usual source of care on HT outcomes [70].

### Implications for Policy

We found an association between reduced co-payments for health care, including for medications, and improved outcomes of HT care in multiple US studies, and in single studies set in Finland, Israel, and Brazil. This is consistent with a wealth of other evidence on how co-payments reduce uptake of necessary care and has clear implications for policy makers, particularly as the balance of evidence does not suggest that reducing medication co-payments leads to an increase in overall health care expenditure [81]–[84]. On balance, we found health insurance coverage to be associated with improved outcomes of HT care in US settings, suggesting that expanded insurance coverage through The Patient Protection and Affordable Care Act (also known as Obamacare) may improve HT outcomes.

### Implications for Research

This study indicates a number of possible implications for future research. Ultimately, an increase in the number of high quality, longitudinal and randomized studies identifying and analyzing the effect of health system arrangements on HT care is required, particularly in LMICs where the majority of the global burden of HT lies, and where weaknesses in health systems are thought to play a significant role in deficiencies in chronic disease care [6]. The focus on financing has highlighted important barriers to effective care and control of HT but needs to be supplemented by research examining other domains, such as delivery and governance mechanisms, production of knowledge, and the social function in the health systems. Most existing studies have a focus on independent effects of different health systems arrangements, thereby creating a “laundry list” of isolated components. Recognizing the shortcomings of this approach, it is important that future studies attempt to capture the complexities and interactions between health systems arrangements. In addition, future national or regional health systems strengthening programs that aim to improve care for chronic conditions such as HT should be robustly evaluated, using longitudinal controlled study designs where possible.

Moving forward, there is a clear need for more robust designs of studies in a much wider range of settings, especially in LMICs. This will ideally include cluster RCTs and prospective longitudinal studies with detailed data on individual and health system characteristics, complemented by qualitative studies to see inside what is often a health systems black box. Such studies also call for consistency in health systems definitions and outcome measures. A particular challenge will be to take account of the complexity of health systems and all health system domains, as well as interpreting studies by not simply as showing what works, but what works in what circumstances [85]. This review should help inform the design of such studies. In particular, the findings are being combined with multi-method appraisals of health systems to understand the barriers faced by patients with HT and their health workers to design cluster randomized trials in several LMICs [86]. Importantly, given that there are many health systems frameworks, this review has shown the practicality of using the one chosen, a framework that is also being used in the multi-method appraisals and that has been found useful in similar previous studies using diabetes as a probe to analyze health systems [12],[13]. Research such as this addresses a crucial gap in understanding of how different models of health systems contribute to health.

## Supporting Information

Table S1 Study designs, settings, findings, and risk of bias of included studies. (DOCX)

Text S1 PRISMA checklist. (DOCX)

Text S2 Search strategy for Medline. (DOCX)

Text S3 Tool for assessing risk of bias for observational studies. (DOCX)

Text S4 Quality appraisal tool for qualitative studies. (DOCX)

## Abbreviations

BP: blood pressure
HT: hypertension
LMIC: low- and middle-income country
mmHg: millimeters of mercury
OR: odds ratio
RCT: randomized controlled trial
RR: relative risk
