# Supplementary material for: The Influence of Health Systems on Hypertension Awareness, Treatment, and Control: A Systematic Literature Review
Source: PLoS Med. 2013 Jul 30;10(7):e1001490. doi: 10.1371/journal.pmed.1001490 (PMC3728036; doi:10.1371/journal.pmed.1001490)
Supplement: Table S1 — (DOCX) [file pmed.1001490.s001.docx]

Table S1: Study designs, settings, findings and risk of bias of included studies.

Key

|  | High risk of bias |
| --- | --- |
|  | Unclear risk of bias |
|  | Low risk of bias |
|  | Complex intervention |

S – Selection bias

D – Differential misclassification bias

N – Non-differential misclassification bias

C – Confounding bias

Abbreviations Used

AOR – adjusted odds ratio

OR – odds ratio

ARR – adjusted risk ratio

SBP – systolic blood pressure

DBP – diastolic blood pressure

BP – blood pressure

SUS - Sistema Único de Saúde (Unified Health System in Brazil)

APR – adjusted prevalence ratio3

USOC – usual source of care

HMO – health maintenance organization

FFS – fee-for-service

| Study (Author, Year, Setting) | Context | Study Design | Sample Size | Health System Domain | Health system arrangement | Outcome | | Relevant findings (95% confidence intervals given in parentheses where available) | | Risk of bias (see key, for details) | | | | | | | |
| --- | --- | --- | --- | --- | --- | --- | --- | --- | --- | --- | --- | --- | --- | --- | --- | --- | --- |
|  |  |  |  |  |  |  |  |  |  | S | | | D | | N | | C |
| Ahluwalia et al., 2010 (USA) [73] | West Virginian women in a screening initiative | Cross-sectional | 733 | Governance and delivery | Routine physician for hypertension care | Control | | Adjusted odds ratio (AOR) of being uncontrolled with a regular physician 0.34 (0.13-0.88) vs. no regular physician (p=0.02) | |  | | |  | |  | |  |
| Ahluwalia et al., 1997 (USA) [36] | Low-income, African-Americans in an urban ambulatory hospital | Case-control | 221 | Health systems financing | Health insurance status | Control | | AOR of being controlled with medical insurance 2.15 (1.02-4.52) vs. no insurance | | low | | |  | | low | | low |
|  |  |  |  |  | Medication costs and co-payments | Control | | AOR of control when medication cost not a deterrent 3.63 (1.59-8.28) vs. cost as a deterrent | |  |  |  |  |  |  |  |  |
|  |  |  |  |  | Co-payments for/costs of medical care | Control | | Odds ratio (OR) of control when cost of care not a deterrent 2.35 (1.19-4.67) vs. no deterrent | |  |  |  |  |  |  |  |  |
|  |  |  |  | Governance and delivery | Routine physician or place of care for hypertension | Control | | OR of control with regular source of care 7.93 (3.86-16.29) vs. no regular source of care | |  |  |  |  |  |  |  |  |
| Ambaw et al., 2012 (Ethiopia) [47] | University hospital with both urban and rural patients | Cross-sectional | 384 | Physical resources | Distance from hospital | Adherence | | AOR of adherence with trip distance <0.5h 2.02 (1.19-3.43) vs. trip distance >0.5hr | | low | | | low | | low | | low |
| Angell et al. 2008 (USA) [56] | Urban population from NYC | Cross-sectional | 1975 | Health systems financing | Health insurance status | Awareness | | Percentage aware with private insurance 86.5% (80.3-90.9), Medicare 85.9% (72.8-93.2; p>0.05), other government insurance 86.9% (77.3-92.8; p>0.05), uninsured 60.2% (46.0-72.8; p<0.05) | | low | | | low | | low | | low |
|  |  |  |  |  |  | Treatment | | Percentage treated with private insurance 76.6% (68.9-82.8), Medicare 81.2% (72.8-93.2; p>0.05), other government insurance 74.5% (64.0-82.8; p>0.05), uninsured 42.6% (28.7-57.7; p<0.05) | |  |  |  |  |  |  |  |  |
|  |  |  |  |  |  | Control | | AOR for control with Medicare 0.92 (0.36-2.33), other government insurance 0.72 (0.30-1.76), uninsured 0.89 (0.30-2.59) vs. private insurance. p>0.05 for all. | |  |  |  |  |  |  |  |  |
|  |  |  |  | Governance and delivery | Routine place of care for hypertension | Awareness | | Percentage aware with a routine place of care 85.1%(80.7-88.6); without a routine place of care 65.5% (51.9-76.9), p<0.05 | |  |  |  |  |  |  |  |  |
|  |  |  |  |  |  | Treatment | | Percentage treated with routine place of care 76.4%(71.3-80.8); without routine place of care 42.1% (30.1-55.1), p<0.05 | |  |  |  |  |  |  |  |  |
|  |  |  |  |  |  | Control | | AOR for control without a routine place of care vs. 0.21 (0.07-0.66) routine place of care, p>0.05 | |  |  |  |  |  |  |  |  |
| Bautista 2008 (USA) [54] | Nationally representative sample | Cross-sectional | 6100 | Health systems financing | Health insurance status | Adherence | | AOR of non-persistence with no health insurance 1.88 (1.24-2.83) vs. with health insurance, p=0.002. | |  | | |  | |  | |  |
| Benkert et al., 2001 (USA) [52] | Urban-Midwest population attending a nurse-managed care centre | Cross-sectional | 52 | Health systems financing | Health insurance status | Control | | Uninsured systolic mean BP 145.703mmHg vs. insured 142.165mmHg (p=0.405). Uninsured mean diastolic BP 85.083mmHg vs. insured 80.483mmHg (p=0.033). | |  | | | low | |  | |  |
| Bleich et al., 2007 (Mexico) [41] | Nationally representative sample | Cross-sectional | 2130 | Human resources | Supply of health professionals | Treatment | | AOR for treatment 1.04(0.85 to 1.26) with high supply of health professionals vs. low supply of health professionals. | | low | | | low | |  | | low |
|  |  |  |  |  |  | Control | | AOR for control 0.81(0.61 to 1.09) with high supply of health professionals vs. low supply of health professionals. | |  |  |  |  |  |  |  |  |
|  |  |  |  | Health systems financing | Health insurance status | Treatment | | AOR for treatment 1.50(1.27, 1.78) Seguro Popular vs. uninsured | |  |  |  |  |  |  |  |  |
|  |  |  |  |  |  | Control | | AOR for control 1.35(1.00, 1.82) Seguro Popular vs. uninsured | |  |  |  |  |  |  |  |  |
| Briesacher et al 2009  (USA) [34] | Nationally representative sample of adults in employment. | Cohort study | 125,937 | Health systems financing. | Medication costs and co-payments | Medication adherence | | AOR for medication adherence vs baseline of 1 for 0$ co-payments. OR = 0.72 (p<0.05) for $1 - 9$ co-payments, OR =1.02 (p>0.05) for $10 - $29 copayments, OR = 1.32 (p<0.05) for co-payments >$30 | |  | | |  | |  | |  |
| Brooks et al., 2010 (USA) [50] | Subgroup of Framingham | Cross-sectional | 1384 | Health systems financing | Health insurance status | Treatment | | Men: OR for treatment 0.19 (0.07-0.56) uninsured vs. insured (p=0.003). Women: odds ratio for treatment 0.31 (0.12-0.79) uninsured vs. insured (p=0.01). | | low | | | low | | low | | low |
|  |  |  |  |  |  | Control | | Men: odds ratio for control 0.17 (0.04-068) uninsured vs. insured (p=0.003). Women: odds ratio for treatment 0.47 (0.16-1.36) uninsured vs. insured (p=0.16). | |  |  |  |  |  |  |  |  |
| Dennison et al., 2007 (South Africa) [44] | Peri-urban black South Africans | Cross-sectional | 403 | Governance and delivery | Private vs. public provision of care | Control | | Non-significant result for association for systolic BP control. DBP 3.29 mmHg greater in public vs. private sector (p=0.042). | |  | | | low | | low | | low |
| DeVore et al., 2010 (USA) [37] | Diverse inner-city population attending tertiary cardiology clinic | Case-control | 154 | Health systems financing | Health insurance status | Control | | OR for BP control for private insurance vs. public insurance = 3.40 (1.25-9.28), p=0.02. | | low | | | low | | low | | low |
| Duru et al., 2007 (USA) [51] | Nationally representative sample | Cross-sectional | 3496 | Health systems financing | Health insurance status | Control | | OR for HT control (amongst hypertensive patients) (ref 1.0 for private insurance), Medicare without private insurance = 0.80 (0.61-1.05, p>0.05), Medicaid 0.75 (0.47-1.20, p>0.05). No insurance 0.63 (0.44-0.92, p=0.05). | | low | | | low | | low | | low |
| Elhayany and Vinker, 2011 (Israel) [25] | Mixed Arab/Jewish patients with low SES from Ramle and Lod | Cohort - before and after study | 260 | Health systems financing | Medication costs and co-payments | Control | | Systolic BP 136.2 (baseline) 137.5 (6 months) 133.0 (12months p =0.04) 128.2 (24 months p<0.001). Diastolic BP 78.0 (baseline) 78.9 (6 months) 75.3 (12months P<0.001) 74.8 (24 moths P<0.001). | | high | | | low | | low | | high |
| Federman et al., 2005 (USA) [48] | All male veteran population | Cross-sectional | 15893 | Human resources | Level of seniority of physician seeing patient | Control | | OR for BP control (baseline =1 for resident). Mid-level doctor 1.12 (0.98-1.28; p>0.05), attending 1.23 (1.08-1.39; p<0.05). | |  | | |  | |  | |  |
| Ford et al., 1998 (USA) [63] | Nationally representative sample | Cross-sectional | 1724 | Health systems financing | Health insurance status | Awareness | | Percent aware: no health insurance 76.1%, Medicaid only 87.2%, other sources of health insurance 78.0% (uninsured vs. Insured= 0.745, Medicaid vs. insured p=0.066, uninsured vs. Medicaid p=0.127). | | low | | | low | | high | | low |
|  |  |  |  |  |  | Treatment | | Percent treated with medication: no health insurance 75.7%, Medicaid only 74.0%, other sources of health insurance 87.8% (uninsured vs. insured p=0.095, Medicaid vs. Insured p= 0.319, uninsured vs. Medicaid p=0.922). | |  |  |  |  |  |  |  |  |
|  |  |  |  |  |  | Control | | Percent treated patients who are controlled: no health insurance 43.1%, Medicaid only 52.3%, other sources of health insurance 39.6% (uninsured vs. Insured p= 0.124, p Medicaid vs. insured p=0.379, uninsured vs. Medicaid p= 0.359). | |  |  |  |  |  |  |  |  |
| Fowler-Brown et al., 2007 (USA) [49] | General population of four US communities in NC, MS, MN, MD | Cohort | 15792 (sub sample unclear) | Health systems financing | Health insurance status | Awareness | | Adjusted Risk Ratio (ARR) for being unaware of a diagnosis of hypertension 1.12 (1.00-1.25) for uninsured vs. insured. | |  | | | low | | low | | low |
|  |  |  |  |  |  | Control | | ARR for inadequate HT control 1.23 (1.08-1.39) for uninsured vs. insured. | |  |  |  |  |  |  |  |  |
| Gai and Gu, 2009 (USA) [26] | Nationally representative sample | Cohort | 3679 | Health systems financing | Health insurance status | Adherence | | OR for medication persistence (baseline 1 for continued private insurance). Continuous public insurance OR =1.324 (0.774-2.266) p=0.304. Multiple coverage gaps OR = 0.636 (0.418-0.969) p=0.035. Uninsured OR = 0.462 (0.282-0.757) p=0.002. | | low | | |  | | low | | low |
| Gandelman et al., 2004 (USA) [68] | General sample of University Medical Centre patients Westchester, NY | Cross-sectional | 614 | Health systems financing | Medication costs and co-payments | Control | | 38% of self-pay or Medicare patients controlled vs. 70 % of Medicaid /privately insured (p<0.001). | |  | | |  | |  | |  |
| Gulliford and Mahabir, 1999 (Trinidad and Tobago) [39] | Diabetic primary care population | Ecological (before and after) | 24 centers | *Human resources | *Annual training workshops for medical officers in Trinidad and Tobago on diabetes care. | Control | | OR for control post intervention vs. pre-intervention = 1.24 (0.84-1.85); p=0.279 | | high | | low | | high | | low | |
|  |  |  |  | *Intellectual resources | *Publication and dissemination of guidelines. |  |  |  |  |  |  |  |  |  |  |  |  |
|  |  |  |  | *Governance and delivery | *National/ regional Audit and evaluation of services |  |  |  |  |  |  |  |  |  |  |  |  |
| He et al., 2002 (USA) [53] | General population | Cross-sectional | 4144 | Health systems financing | Health insurance status | Control | | OR for control (baseline no insurance): government insurance = 1.08 (0.70-1.68; p=0.72); private insurance = 1.59 (1.02-2.49; p=0.04). | | low | | low | | low | | low | |
|  |  |  |  | Governance and delivery | Routine place of care for hypertension | Control | | OR for control same health facility of care 2.77 (1.88-4.09) vs. lack of same facility of care (p<0.001) | |  |  |  |  |  |  |  |  |
|  |  |  |  |  | Routine physician for hypertension care | Control | | OR for control same health provider of care 2.29 (1.74-3.02) vs. lack of same provider of care (p<0.001) | |  |  |  |  |  |  |  |  |
| Hill et al., 2002 (USA) [57] | Inner-city black men presenting to the emergency department | Cross-sectional | 309 | Health systems financing | Health insurance status | Control | | Non-significant association between health insurance status and BP control, magnitude of association not reported in paper. | |  | | low | | low | | low | |
|  |  |  |  | Governance and delivery | Routine physician for hypertension care | Control | | Non-significant association between regular MD for HT care and BP control, magnitude of association not reported in paper. | |  |  |  |  |  |  |  |  |
| Hsu et al., 2006 (USA) [27] | Subset belonging to Kaiser Permanente in Northern California | Cohort | 104948 | Health systems financing | Medication costs and co-payments | Control | | OR for poor control = 1.05(1.00-1.09) in capped vs. uncapped drug benefits | | low | | low | | low | | low | |
|  |  |  |  |  |  | Adherence | | OR for drug non adherence with cap on drug benefits OR = 1.30 (1.23-1.38) vs. no cap | |  |  |  |  |  |  |  |  |
| Hyman and Pavlik, 2001 (USA) [58] | Nationally representative sample | Cross-sectional | 10576 | Health systems financing | Health insurance status | Awareness | | AOR for lack of awareness of hypertension (HT): has health insurance 0.91 (0.61-1.34) vs. has no health insurance (p=0.62) | | low | low | | | | low | | low |
|  |  |  |  |  |  | Control | | AOR for acknowledged uncontrolled HT: has health insurance 1.30 (0.79-2.13) vs. has no health insurance (p=0.29) | |  |  |  |  |  |  |  |  |
|  |  |  |  | Governance and delivery | Routine place of care for hypertension | Awareness | | AOR for lack of awareness of HT: has usual source of care: 1.12 (0.87-1.43) vs. has no usual source of care (p=0.38) | |  |  |  |  |  |  |  |  |
|  |  |  |  |  |  | Control | | AOR for acknowledged uncontrolled HT: has usual source of care: 1.07 (0.63-1.84) vs. has no usual source of care (p=0.79). | |  |  |  |  |  |  |  |  |
| Jokisalo et al., 2002 (Finland) [67] | Nationally representative sample | Cross-sectional | 1561 | Health systems financing | Medication costs and co-payments | Adherence | | % non-compliant with: a low lack of special reimbursement for medication (12%); a medium lack of special reimbursement for medication (25%); a high lack of special reimbursement for medication (24%); p<0.001 between groups. | | high | low | | | |  | | high |
| Kang et al., 2006 (USA) [59] | Low SES Korean-American elderly | Cross-sectional | 146 | Health systems financing | Health insurance status | Treatment | | AOR for treatment 2.41 (0.91-6.39) any insurance vs. no insurance. AOR for treatment Medicare vs. no insurance = 2.06 (0.66-6.42). AOR for treatment Medicaid vs. no insurance = 3.21 (0.89-11.61). AOR for treatment private insurance vs. no insurance = 1.46 (0.29-7.39) | | high | low | | | |  | | high |
|  |  |  |  |  |  | Control | | No significant univariate associations between insurance type and HT control (p>0.05). | |  |  |  |  |  |  |  |  |
| Keeler et al., 1985 (USA) [24] | Nationally representative sample, subset of RAND study | RCT | 3958 | Health systems financing | Co-payments for/costs of medical care | Control | | Adjusted mean difference in diastolic BP (free plan - cost sharing plans) All patients (N=3495) -0.9 (-1.6,-0.2) p<0.05. Hypertensive patients (N=856) = -1.9 (-3.5, -0.3) P<0.05. Adjusted mean difference in systolic BP (free plan -cost sharing plan) All patients (N=3495) -0.7 (-1.6, 0.2) P>0.05. Hypertensive patients (n=856) -1.8 (-4.5, 0.6) P>0.05 | High risk of participant and personnel blinding. Unclear risk of random sequence generation, allocation concealment, and blinding of outcome assessment. Low risk of selective reporting and incomplete outcome data. | | | | | | | | |
| Khosravi et al., 2010 (Iran) [38] | General population sample from University Hospital | Ecological (before and after) | Before: n=6175 (intervention area), N=6339 (reference area). After: n=4719 (intervention area) n=4853 (reference area) | *Human resources | *Continuous medical education (CME), establishing educational cores, and publishing educational books and local guidelines. | Awareness | | Aware (%). Intervention area before = 40.4, after = 49.8 (p<0.001). Reference area before = 41.7, after = 46.7 (p=0.021) | | low | | |  | |  | | high |
|  |  |  |  | *Intellectual resources | *Publishing educational books and local guidelines healthcare providers | Treatment | | Treated (%). Intervention area before = 34.9, after = 43.8 (p<0.001). Reference area before = 35.4, after = 40.0 (p=0.031) | |  |  |  |  |  |  |  |  |
|  |  |  |  | *Governance and delivery | *Provision of free BP screening and risk assessment | Control | | Controlled (%). Intervention area before = 7.1, after = 15.8 (p<0.001). Reference area before = 9.8, after = 14.0 (p=0.003) | |  |  |  |  |  |  |  |  |
| Kotchen et al., 1998 (USA) [75] | Inner-city African American population from Milwaukee | Cross-sectional | 583 | Governance and delivery | Private vs. public provision of care | Control | | OR for HT control with private provider 1.20 (0.62-2.32) vs. non-private provider. | |  | | |  | |  | |  |
| Labhardt et al., 2010 (Cameroon) [28] | Rural population attending primary care clinic | Cohort - before and after study | 493 | *Physical resources | *Sphygmomanometers, stethoscopes provided to health centers. Basic medications added to pharmacy stocks. | | Control | Fall in systolic BP baseline to follow up : -26.5mmHg (-12.5 to -40.5): diastolic BP -17.2mmHg (-7.1 to -27.3) | | high | | | high | | low | | low |
|  |  |  |  | *Human resources | *Task shifting from physicians in the hospitals to Non-Physician clinicians. | |  |  |  |  |  |  |  |  |  |  |  |
|  |  |  |  | *Intellectual resources | *Treatment protocols were adapted from international recommendations to local conditions. | |  |  |  |  |  |  |  |  |  |  |  |
|  |  |  |  | *Governance and delivery | *National/ regional audit and evaluation of services | |  |  |  |  |  |  |  |  |  |  |  |
| Li et al., 2012 (USA) [29] | Nationally representative sample | Cohort | 54594 | Health systems financing | Medication costs and co-payments | Adherence | | AOR for non-adherence (less than 80% days covered by prescriptions) - baseline =1 (low income subsidy-no drug coverage gap). Brand name and generic gap coverage OR = 1.00 (0.88 to 1.15), Generic gap coverage OR = 1.50 (1.30 to 1.73). No gap coverage OR = 1.60(1.50 to 1.71). | |  | | |  | |  | |  |
| Maciejewski et al., 2010 (USA) [30] | Veterans from Veteran Affairs Medical Centers | Cohort | 7090 | Health systems financing | Medication costs and co-payments | Adherence | | Year after copayment increase: difference in adherence = -1.8% (-1.8, -1.9) in co-payers compared to exempt controls. 2 years after copayment increase: difference in adherence = -3.2% (-3.1,- 3.3) in co-payers compared to exempt controls. | | low | | | low | | low | | low |
| Mbouemboue et al 2012  (Cameroon) [46] | Mixed rural and urban sample in Adamawa Region | Cross-sectional | 117 | Health systems finance | Medication costs and co-payments | Awareness | | OR for HT awareness (baseline 1 for low cost of medications): medium cost 0.35 (0.06 - 2.07), high cost 0.44 (0.07 - 2.75) | |  | | |  | |  | |  |
| Mejia-Rodriguez et al., 2009 (Mexico) [43] | Patients from family medicine units in Morelia and Michoacán | Cross-sectional | 4040 | Human resources | Treated by specialist physician vs. general practitioner | Control | | OR for uncontrolled hypertension in those treated by non-specialists vs. specialists 1.43 (1.20-1.71) | | low | | | low | |  | | low |
| Moy et al., 1995 (USA) [55] | Nationally representative sample | Cross-sectional | 6158 | Health systems financing | Health insurance status | Treatment | | AOR for no treatment (reference 1 for any private insurance), Medicare or Medicaid AOR = 1.19 (0.99-1.41), Uninsured AOR = 1.49 (1.18-1.89) | | low | | |  | | high | | low |
|  |  |  |  | Governance and delivery | Routine place of care for hypertension | Treatment | | AOR for no treatment (reference 1 for physician's office) Clinic AOR = 1.07 (0.90-1.28), Emergency department AOR = 1.36 (0.73-2.55), No usual place of care AOR = 3.94 (3.05-5.08) | |  |  |  |  |  |  |  |  |
|  |  |  |  |  | Routine physician for hypertension care | Treatment | | AOR for no treatment (reference 1 for general or family practitioner), Internist AOR = 0.82(0.67-1.00), Non primary care physician AOR = 1.20(0.97-1.49), No particular physician AOR = 2.61 (2.15-3.18) | |  |  |  |  |  |  |  |  |
| Nguyen et al., 2011 (USA) [64] | Population sample from NYC | Cross-sectional | 1334 | Health systems financing | Health insurance status | Awareness | | awareness OR= 1.2 (0.4-4.1) public vs. private | | low | | | low | | low | | low |
|  |  |  |  |  |  | Treatment | | public insurance OR = 1.1 (0.4-3.6) vs. private insurance | |  |  |  |  |  |  |  |  |
|  |  |  |  |  |  | Control | | Average SBP 6.7mmHg lower with private insurance vs. public (p=0.04) | |  |  |  |  |  |  |  |  |
|  |  |  |  | Governance and delivery | Routine place of care for hypertension | Awareness | | Awareness: OR= 1.0(0.2-5.6) no usual care vs. usual place of care (baseline) | |  |  |  |  |  |  |  |  |
|  |  |  |  |  |  | Treatment | | Treatment OR= 0.2 (0.1-0.8) no usual care vs. usual place of care (baseline) | |  |  |  |  |  |  |  |  |
|  |  |  |  |  |  | Control | | SBP 16.4 mmHg higher with no usual place of care (p=0.02). | |  |  |  |  |  |  |  |  |
| Nissinen et al., 1983 (Finland) [31] | Community study in North Karelia | Cohort - before and after study | 3002 | *Human resources | *Training of health personnel | Control | | Blood pressure levels fell further in both hypertensive men and women in intervention region compared to control region (p<0.001). | | high | | |  | | low | | high |
|  |  |  |  | *Intellectual resources | *Creation of a new information system |  |  |  |  |  |  |  |  |  |  |  |  |
|  |  |  |  | *Governance and delivery | *Integration of hypertension care into existing primary care services |  |  |  |  |  |  |  |  |  |  |  |  |
| Pesa et al., 2012 (USA) [32] | Nationally representative sample | Cohort | 26688 | Health systems financing | Medication costs and co-payments | Adherence | | For every US$1.00 increase in cost sharing, proportion of days covered (PDC) decreased by 1.1 days (p<0.0001). | |  | | |  | |  | |  |
| de Santa-Helena et al., 2010 (Brazil) [42] | Patients from family health units in Blumenau | Cross-sectional | 595 | Health systems financing | Medication costs and co-payments | Adherence | | AOR for non adherence 4.9 (1.6-15.3) for those who need to pay for medications vs. those who have drugs provided by Sistema Único de Saúde (SUS – Unified Health System); p<0.05 | | low | | | low | |  | | low |
|  |  |  |  | Governance and delivery | Private vs. public provision of care | Adherence | | AOR for non adherence 1.8 (1.1-2.7) SUS vs. private medical provider; p<0.05. | |  |  |  |  |  |  |  |  |
| Schoen et al., 2001 (USA) [65] | Uninsured patients at an inner-city University-based outpatient clinic | Cohort | 137 | Health systems financing | Medication costs and co-payments | Control | | Percent of uncontrolled hypertensive pateints reaching therapeutic goal increased from 19.0% at baseline to 36.8% at 6 months (p<0.001) and 65.8% at 24 months (p<0.01). | |  | | |  | |  | |  |
| Shea et al., 1992a (USA) [35] | Cases: severe uncontrolled hypertension or hypertensive encephalopathy or hypertensive emergency. Controls: hypertensives w/o severe uncontrolled hypertension in inner-city NYC | Case-control | 207 | Health systems financing | Health insurance status | Control | | AOR for severe uncontrolled HT 1.9 (0.8-4.6) without insurance vs. with insurance (p=0.16). | | low | | |  | |  | | low |
|  |  |  |  | Governance and delivery | Routine physician for hypertension care | Control | | AOR for severe uncontrolled HT 3.5 (1.6-7.7) with no primary care physician vs. having a primary care physician (p=0.003) | |  |  |  |  |  |  |  |  |
| Shea et al., 1992b (USA) [60] | Hospital-based African American and Hispanic inner-city population in NYC | Cross-sectional | 207 | Health systems financing | Health insurance status | Adherence | | Health insurance was not significantly associated with adherence in a multivariable model (numbers were not reported) | |  | | |  | |  | |  |
|  |  |  |  | Governance and delivery | Routine physician for hypertension care | Adherence | | AOR for non adherence 2.9 (1.36, 6.02) for lack of primary care physician vs. presence of primary care physician (p<0.01). | |  |  |  |  |  |  |  |  |
| Spatz et al., 2010 (USA) [70] | Nationally representative sample | Cross-sectional | 6762 | Governance and delivery | Routine place of care for hypertension | Treatment | | APR (adjusted prevalence ratios) for being untreated = 2.43(1.88-2.85) for no usual source of care (USOC) vs. USOC. Joint effects for insurance and USOC: APR for being untreated: No USOC and insured APR=2.58 (1.87-3.08), USOC yes and uninsured APR = 1.97 (1.38-2.52), No USOC and uninsured APR = 3.06 (2.37-3.39) | | low | | | low | | low | | low |
| Tu et al., 2009 (Canada) [40] | Primary Care | Ecological (cross-sectional) | 135 | Health systems financing | Physician remuneration model | Awareness | | Screening (detection) 1 BP visit in last 3 years: salary 93.5%, fee for service 93.3%, capitation 90.6% (p=0.22 between groups) | |  | | |  | | low | | low |
|  |  |  |  |  |  | Treatment | | Treatment (at least 1 medication) salary 81.0%, fee for service 87.4%, capitation 90.9% (p=0.01 between groups). | |  |  |  |  |  |  |  |  |
|  |  |  |  |  |  | Control | | Control (last reading below target) salary 38.6%, fee for service 41.6% capitation 54.5% (p<0.01 between groups) | |  |  |  |  |  |  |  |  |
| Turner et al., 2009 (USA) [62] | Mostly black women in Philadelphia | Cross-sectional | 300 | Health systems financing | Health insurance status | Adherence | | AOR for adherence to antihypertensives: In the past year had to go without usual BP medications because not covered (yes) 1.29 (0.26-9.49) vs. no (p=0.76). | | high | | | low | | low | | low |
| Udvarhelyi et al., 1991 (USA) [69] | Patients from facility where physicians are HMO and FFS | Cross-sectional | 246 | Health systems financing | Physician remuneration model | Control | | AOR for hypertension control = 1.82(1.02 to 3.27) for health maintenance organization (HMO; reimbursed through capitation) vs. fee-for-service (FFS) patients. | |  | | | low | |  | | low |
| Victor et al., 2008 (USA) [72] | Mostly non-Hispanic blacks from Dallas County | Cross-sectional | 1514 | Governance and delivery | Routine physician for hypertension care | Awareness | | AOR for awareness 3.81 (2.86-5.07) has a regular physician vs. has no regular physician. | | low | | | low | | low | | low |
|  |  |  |  |  |  | Treatment | | AOR for treatment 8.36 (5.95-11.74) has a regular physician vs. has no regular physician. | |  |  |  |  |  |  |  |  |
|  |  |  |  |  |  | Control | | AOR for control 5.23 (3.30-8.29) has a regular physician vs. has no regular physician. | |  |  |  |  |  |  |  |  |
| Wong et al., 2010 (Hong Kong, China) [33] | Chinese patients in primary care | Cohort | 83884 | Health systems financing | Co-payments for/costs of medical care | Adherence | | AOR for adherence fee payers 1.14 (1.09 to 1.19) vs. fee waivers (p<0.001). | |  | | |  | |  | |  |
| Wyatt et al., 2008 (USA) [61] | African American population from Jackson, MS | Cross-sectional | 4986 | Health systems financing | Health insurance status | Adherence | | Non-significant association (p>0.05), measure of association only reported for significant predictors. | |  | | | low | | low | | low |
|  |  |  |  |  |  | Treatment | | Non-significant association (p>0.05), measure of association only reported for significant predictors. | |  |  |  |  |  |  |  |  |
|  |  |  |  |  |  | Control | | Non-significant association (p=0.058), p value reported in discussion, measures of association (odds ratios) only reported for significant predictors. | |  |  |  |  |  |  |  |  |
| Yiannakopoulou et al., 2005 (Greece) [74] | Patients admitted for elective surgery in Athens. | Cross-sectional | 1000 | Governance and delivery | Private vs. public provision of care | Adherence | | Compliance with private physician 25.1% versus 10% of those with physician in rural areas and 8.8% of with physician from the National Health System (p<0.005 between groups). | |  | | |  | |  | |  |
| Yoon and Ettner, 2009 (USA) [66] | Generally representative US sample, all insured. | Cross-sectional | 83893 | Health systems financing | Medication costs and co-payments | Adherence | | Effect of co-payments on medication adherence stratified by baseline level of adherence to medication. Amongst people with low to median baseline levels of adherence to medication (10th, 25th and 50th centile) increased co-payments had a significant negative effect on adherence to anti-hypertensive medication. Regression coefficients for adherence amongst different subgroups according to baseline adherence as follows: 10th centile of adherence. (Co-payment <5$ = baseline). Co-payment 6-12$ β=-7.96 (p<0.01). Co-payment>15$ β=-9.13 (p<0.01). 25th centile of adherence (Co-payment <5$ = baseline). Co-payment 6-12$ β =-5.96 (p<0.01). Co-payment>15$ β =-5.88 (p<0.01). 50th centile of adherence (Co-payment <5$ = baseline). Co-payment 6-12$ β =-2.92 (p<0.01). Co-payment>15$ β =-2.21 (p<0.01). 75th centile of adherence (Co-payment <5$ = baseline). Co-payment 6-12$ β =0.29 (p>0.01). Co-payment>15$ β =-0.10 (p>0.01). 90th centile of adherence (Co-payment <5$ = baseline). Co-payment 6-12$ β =3.13 (p<0.01). Co-payment>15$ β=1.28 (p>0.01). | | Low | | | Low | | Low | |  |
| Yu et al 2013  (China) [45] | China. Low income rural residents in Shandong province. | Cross-sectional | 204 | Health systems financing | Medication costs and co-payments | Adherence | | Improved medication adherence in free-medication group compared to fee payers(p=0.034) | |  | | |  | |  | |  |
|  |  |  |  |  |  | Treatment | | 0% of free-medication group not taking any medication compared to 14.7% in fee payers (p<0.001) | |  |  |  |  |  |  |  |  |
|  |  |  |  |  |  | Control | | 12.7% in free medication group vs. 11.8% in fee payers have controlled hypertension. P=0.831 | |  |  |  |  |  |  |  |  |
