# Supplementary material for: The Influence of Health Systems on Hypertension Awareness, Treatment, and Control: A Systematic Literature Review
Source: PLoS Med. 2013 Jul 30;10(7):e1001490. doi: 10.1371/journal.pmed.1001490 (PMC3728036; doi:10.1371/journal.pmed.1001490)
Supplement: Text S2 — (DOCX) [file pmed.1001490.s003.docx]

Text S2: Search Strategy used for Medline via Ovid

1 *hypertension/ (130173)

2 *blood pressure/ (60894)

3 ((high or increased or elevated) adj2 blood pressure).ab,ti. (21372)

4 ((high or increased or elevated) adj2 (diastolic or systolic or arterial)).ab,ti. (12359)

5 (pre?hypertens* or hypertens*).ab,ti. (280247)

6 ('systolic blood pressure' or 'diastolic blood pressure').ab,ti. (44494)

7 1 or 2 or 3 or 4 or 5 or 6 (369614)

8 "equipment and supplies"/ (17450)

9 *health facilities/ (6515)

10 *blood pressure determination/ (7840)

11 *health services administration/ (2868)

12 *health education/ (28213)

13 *delivery of healthcare/ (36669)

14 *primary healthcare/ (30772)

15 *internet/ (22844)

16 *health services accessibility/ (21661)

17 *health planning/ (11383)

18 *health priorities/ (3864)

19 *"fees and charges"/ (3268)

20 *insurance coverage/ (3890)

21 *health services research/ (11893)

22 *health status disparities/ (3506)

23 *social support/ (15824)

24 *financing, government/ (7255)

25 8 or 9 or 10 or 11 or 12 or 13 or 14 or 15 or 16 or 17 or 18 or 19 or 20 or 21 or 22 or 23 or 24 (223707)

26 25 and 7 (5538)

27 (Diagnos* adj3 equipment).ab,ti. (661)

28 (Health* adj2 (Facilities or facility)).ab,ti. (10135)

29 Health record system*.ab,ti. (211)

30 (Mobile unit* or Sphygmomanometer or Consumable* or Ambulatory blood pressure measurement or (Monitoring adj2 equipment)).ab,ti. (3488)

31 ((Drug* or pharmaceutical* or medicine* or Medicat*) adj3 (Availability or affordability or appropriat* or cost*)).ab,ti. (15395)

32 ((Staff* or worker*) adj3 (train* or educat* or healthcare or model)).ab,ti. (16573)

33 (Provide* adj1 educat*).ab,ti. (2089)

34 (Polic* adj1 (maker* or planner* or manager*)).ab,ti. (8288)

35 ((Inform* or empower*) adj2 (patient* or user* or consumer* or staff)).ab,ti. (22259)

36 national guid*.ab,ti. (3442)

37 (Inform* adj2 (self-care or healthy lifestyle* or diet* or physical activit*)).ab,ti. (1699)

38 (Standard* adj2 (enforce* or implement* or monitor*)).ab,ti. (2731)

39 "Rational drug use".ab,ti. (170)

40 (Community adj3 (organisation* or mobili?ation)).ab,ti. (716)

41 ((Consumer or Stakeholder) adj2 participation).ab,ti. (316)

42 "Clinical governance".ab,ti. (886)

43 (Leadership or centrali* or decentrali*).ab,ti. (30927)

44 ("policy authority" or (management adj2 approach*) or "quality assurance" or (setting adj2 practice) or "Chronic Care Model").ab,ti. (25459)

45 (manag* adj2 (capacity or performance)).ab,ti. (1121)

46 ((Legal or administrat*) adj2 framework).ab,ti. (742)

47 (Integrat* adj2 care).ab,ti. (3681)

48 ((System or policy) adj2 design).ab,ti. (1985)

49 (Taxation or "Targeted payment" or "Targeted payments" or Commissioning or Capitation or Cost?sharing or Financing).ab,ti. (13141)

50 (Fee adj2 service).ab,ti. (3008)

51 (social adj2 (capital or support)).ab,ti. (19348)

52 ((Social?health or Community?based health or Private) adj3 insurance).ab,ti. (3267)

53 27 or 28 or 29 or 30 or 31 or 32 or 33 or 34 or 35 or 36 or 37 or 38 or 39 or 40 or 41 or 42 or 43 or 44 or 45 or 46 or 47 or 48 or 49 or 50 or 51 or 52 (181938)

54 53 and 7 (4582)

55 26 or 54 (9367)

56 ((Diagnos* or aware* or detect* or ident* or treat* or manag* or control* or adher* or screen*) adj3 (blood pressure or hypertens* or diastolic or systolic)).ab,ti. (63592)

57 ((medication or drug or treatment) adj3 (compliance or adherence)).ti,ab. (15444)

58 exp medication adherence/ (4576)

59 exp patient compliance/ (47845)

60 56 or 57 or 58 or 59 (116677)

61 55 and 60 (2949)

62 (animals not (humans and animals)).sh. (3688338)

63 (rat or rats or rodent* or mouse or mice or murine or dog or dogs or canine* or cat or cats or feline* or rabbit or rabbits or pig or pigs or porcine or swine or sheep or ovine* or guinea pig*).ti. (1543224)

64 62 or 63 (3904407)

65 61 not 64 (2923)
