# Supplementary material for: The Influence of Health Systems on Hypertension Awareness, Treatment, and Control: A Systematic Literature Review
Source: PLoS Med. 2013 Jul 30;10(7):e1001490. doi: 10.1371/journal.pmed.1001490 (PMC3728036; doi:10.1371/journal.pmed.1001490)
Supplement: Text S3 — (DOCX) [file pmed.1001490.s004.docx]

Text S3. Tool for assessing risk of bias for observational studies

| **Type of bias** | **Study design** | | | |
| --- | --- | --- | --- | --- |
|  | **Cross sectional** | **Case control** | **Cohort** | **Ecological** |
| Selection bias | Was the study population selected appropriate? | | | |
|  | Was the sample representative of its target population? | Were the controls randomly selected from the same population as the cases? | Was an appropriate control group used?  Was follow up sufficiently complete? (>80%) | Were the subjects representative of the group, place, or population of interest? |
| Differential misclassification | Did the assessment of the exposure or outcome differ according to the patient status? | Did the exposure assessment differ for cases and controls? | Did the outcome assessment differ for exposed and non exposed? | Were the exposure and outcome variables measured and defined in the same or a similar way across the different groups studied? |
| Non-differential misclassification | Were valid methods used for measuring hypertension awareness, treatment or control and medication adherence? | | | |
| Confounding | Was any strategy undertaken to control for potential confounders?   1. At the design stage (restriction, matching) 2. At the analysis stage (stratification, multivariable analysis) | | | |

**Define each domain as low risk of bias, unclear risk of bias or high risk of bias**
